# Abstracts of papers presented at the ISLAR (International Symposium on Laboratory Automation and Robotics) 1996

**DOI:** 10.1155/S1463924697000102

**Published:** 1997


Journal of Automatic Chemistry, Vol. 19, No. 3 (May-June 1997) pp. 61-89

### Abstracts of papers presented at the ISLAR (International Symposium on Laboratory Automation and Robotics) 1996

The fourteenth International Symposium on Laboratory Automation and Robotics was held from 20-23 October 1996 in Boston, MA, USA, and proved the largest number of technical presentations ever offered at this meeting. State-of-the art developments in laboratory automation and robotics were reflected in the symposium programme, including papers and posters on all aspects of the technology—drug discovery research, data handling and data management, chemical analysis, custom automation, laboratory workstations, bioanalytical assays, managing laboratory automation, dissolution testing, pharmaceutical analysis, automation and combinatorial chemistry, validating automated methods, and advanced topics. Several new workshops and discussion sessions were also provided.

Zymark should be congratulated on their continuing commitment to this programme of seminars and technical sessions. The programme provided a valuable insight to the use of robotics as well as the problems experienced. We hope to include some of these articles as technical papers in the journal in the near future.

The 1997 ISLAR will once again be held in Boston, MA, from 19-22 October 1997. Speakers' abstracts should be submitted by 14 March and Poster abstracts by 14 July. Session topics will be similar to previous years but will also include High Throughput Screening, Re-Engineering the Laboratory and Increasing Productivity. For more information, contact Christine O'Neil at 508 497 2224; fax on 508 435 3439; send an E-mail to islar@ISLAR.com or visit the ISLAR pages at http://www.islar.com.

### Drug discovery meets biotech: how automation enables the 'better-faster-cheaper' paradigm

**Walter H. Moos**
*Chiron Corporation, Emeryville, CA, USA*

Pharmaceutical and biotechnology companies have faced significant new challenges in the 1990s. Health care cost-containment, mergers and acquisitions, venture-capital funded technology development, and other forces have catalysed important paradigm shifts in therapeutic research and development. The resulting efforts to perform drug discovery and preclinical development better, faster, and cheaper, are being aided by new approaches in the design, synthesis, screening, and optimization of product candidates. Core competencies in automation underpin a major portion of this new wave of unprecedented productivity. Several examples of Chiron's cluster of combinatorial technologies were discussed.

### The strategic direction of bioanalytical support for drug development

**James E. McClurg**
*Harris Life Science, Lincoln, NE, USA*

The types and volume of bioanalytical support of drug development being outsourced by pharmaceutical companies is changing rapidly. There are a number of forces causing this change: bioanalytical technology, cost and time pressures, the proportion of drug development being done by 'start-up' ventures, compliance issues, geographic locations, sample logistics, clinical study designs, types of therapeutic molecules, competition, use of toxicokinetic data to design early clinical trials, etc.

These issues and the roles they play in painting the opportunities/challenges picture of outsourced bioanalytical support for drug development on the five year horizon were discussed.

### The automated synthesis of organic compounds—some newcomers have some success

**Christopher Floyd, Christopher Lewis, Sanjay Patel and Mark Whittaker**
*British Biotech Pharmaceuticals Ltd, Oxford, UK*

The search for useful drug substances is accelerating. The increasing speed and reliability of automated assays using microtitre plates has led to a situation where traditional one-by-one organic synthesis can no longer keep up, and automated devices are required for organic synthesis, purification, and distribution for assay. By deliberately avoiding over-automation the authors have a flexible system in which the devices can be used in the most appropriate way, as different therapeutic project areas have different, and changing, needs. In particular they work at a scale that provides enough material for follow-up *in vivo* testing, as well as initial screening.

A key part of the system is a Zymark XP Zymate laboratory robot. It is equipped with custom-built work stations to enable it to cleave small molecules into solution after solid phase synthesis, to evaporate the acidic solutions to near dryness, and to dilute, dissolve and distribute into 96-well plates. All of these functions may be used independently, or chained together for a complete processing run. A MultiSyn Tech Syro I is used for the solid phase syntheses and a Gilson Preparative HPLC using an Aspec XL as an autoinjector and fraction collector is used for purification, if required.

0142-0453/97 $12·00 © 1997 Taylor & Francis Ltd

The presentation described the synthesis and subsequent treatment of some sulphonamides and small peptides, demonstrating how some of the disparate needs of the different projects were met.

### Acclerating drug discovery by high-throughput combinatorial synthesis

**Steven C. Banville and Ronald N. Zuckermann**
*Chiron Corporation, Emeryville, CA, USA*

A fully automated organic synthesizer has been constructed to generate diverse chemical libraries for use in drug screening programmes. This high-throughput chemical synthesizer is based on proprietary robotic synthesis hardware and software. The robotic synthesizer is able to transform a small set of low-cost starting materials into an equimolar mixture of tens of thousands of novel diverse chemical structures. These mixtures can then be screened against a wide variety of pharmaceutically relevant receptors. Reptoids (N-substituted glycines), as well as peptides, can be synthesized in very high yields. Peptoids have the added advantage that they can be synthesized from àn incredibly large pool of commercially available primary amines. Highly potent peptoid trimers have been discovered with this technology. More complex chemical structures are also being made; this has prompted significant design changes in the synthesis hardware. Recently, a new generation synthesizer has been constructed that features a high temperature heat block as well as more flexible software. This automated system has thus evolved from peptide synthesizer into a more general purpose organic synthesizer.

### Automated organic chemical synthesis at Organon

**P. Hilberink, S. v. Aelst, F. Kaspersen**
*Scientific Development Group, N.V. Organon, The Netherlands*
**T. Vink**
*E/I/A Automation, N.V. Diosynth, The Netherlands*
**and E. v. Gool**
*Labotec SA/NV, Teralfene, Belgium*

This presentation described Organon's synthesis robot which is used for reaction optimization for the synthesis of biologically active compounds. Some results of the experiments performed with this system were also presented. The automated synthesis system is centred around a Zymark XP-robot. With the use of a number of working stations (vortex; capping-station, cleaning station, solvents-station, balance), storage racks for reagents/samples and four different robot-hands, the system is capable of performing (this means starting, actual chemical reaction, sampling, partial work up and cleaning) automatically a great variety of organic reactions in two reaction vessels. The reaction vessels have a working volume of 25–125 ml (so experiments on preparative scale can be done), the temperature can be varied from −40°C to 140°C (reflux is possible) in either $N_2$ or Ar-atmosphere with controlled stirring. The system is set up in a vented hood and the power supply is backed up by a battery pack. Control of the robot and the reaction vessels is separated by the use of two System V controllers. In case of a robotic error, it is always possible to control the reaction vessels. Programming is through a software program developed in house, running on a standard 486 computer. The recipes for reactions are constructed from unit operations where variables like temperature, reaction time can be introduced.

The automated synthesis system has been operational since January 1995. Since then some 250 reactions have been done successfully; some typical examples in the field of steroid/carbohydrate/heterocyclic chemistry were shown. From these experiments some drawbacks of the synthesis system could be identified; improvements that will be/have been introduced, were discussed.

### Adaptation of liquid handlers to the automation of organic and combinatorial chemistry

**William Neil, Harold N. Weller, Michael Lawrence and Marian G. Young**
*The Bristol-Myers Squibb Pharmaceutical Research Institute, Princeton, NJ, USA*

High throughput screening has been at the forefront of laboratory automation for several years, resulting in rapid and efficient biological testing. Recent efforts have focused on automating the synthesis of new candidate molecules for screening, particularly through the use of combinatorial methods. Traditional robotic liquid handlers have been designed for delivery of aqueous samples for biological assays, and are not necessarily suitable for delivery of organic solvents and solutes. Additionally, screening applications are often run by a dedicated operator or team of operators, while apparatus for combinatorial organic synthesis may be used by a large and heterogeneous group often located at more than one site. Several of the problems and solutions related to conversion of conventional robotic liquid handlers to use in organic synthesis were discussed.

The Hamilton Microlab 2200 liquid handler is used by the authors to perform many liquid handling tasks, including product distribution, and reagent delivery for both solution and solid phase chemistry. Many hardware and software modifications have been incorporated into the system to adequately support the needs of organic and combinatorial chemistry. By using a universal deck layout and common carriers identical systems can be provided at several sites. The methods are not specific to the Hamilton Microlab, and application to other liquid handlers was discussed.

### Managing laboratory automation—historical retrospective and future view

**Keith D. Holmes**
*Pfizer Inc., Groton, CT, USA*

A retrospective view of automation and management from the late 1970s until today shows that industry and instrument companies have made tremendous strides in automation. However, the management of laboratory automation in future will probably be dramatically different from today's management. The 'virtual laboratory' may be a real possibility and rapid chemical screening of hundreds of compounds a month may be commonplace. The relationship between industry and academia (at all levels) will become much more collaborative. In this presentation, the author discussed the preparations that might be made to meet these challenges.

### Relocating and re-establishing a robotics laboratory

**Stephen Scypinski, Theodore Sadlowski and John Baiano**
*Hoffmann-La Roche Inc., Nutley, NJ, USA*

Over the past several years, there have been many presentations at various ISLAR meetings devoted to the trials and tribulations of establishing a robotics facility in an existing laboratory environment. The considerations necessary to properly fit out such a laboratory have been discussed. Recently, the Analytical R&D Department at Hoffmann-La Roche has had the good fortune (or painful task) or moving out of an existing laboratory into a newly constructed building. The new laboratory was specifically designed to accommodate robotic systems and was not 'converted' from a laboratory already in use. This allowed the greatest efficiency in the use of space and in design of services needed for the systems. It was also a learning experience, with regard to areas such as building, utility and municipal codes. The room was even fitted with its own high-pressure hot water system to supply the cleaning stations on the robots.

Once the laboratory had been completed and a certificate of occupancy was obtained, the robotic systems had to be moved. The monumental task of disassembling, packing, moving and then reassembling three Py systems, as well as the various workstations along with their supporting instrumentation (HPLC pumps and detectors), took several months to completely accomplish. After reinstallation, the process of revalidation was undertaken.

This presentation offered tips and information to anyone faced with, or contemplating, moving their robotic systems to a new location.

### Laboratory automation—successful approach

**Myriam L. Martinez**
*McNeil Consumer Products, Las Piedras, PR, USA*

McNeil Consumer Products (PR) Inc. is a manufacturing site for solid dosages of Tylenol (all adult products), Children Chewables, Tylenol PM and Tylenol Sinus. The Analytical Laboratory is under quality assurance and is responsible for the release of all products after testing. An important factor is that it works in a 'just in time' environment, where the releases have to be done immediately to have the product always available in the market. The initial approaches for the acquisition of automated systems were to release Tylenol PM products as quickly as possible without adding headcount. When the new equipment was installed and running the following resulted.

(1) Automation allowed the product to be released in less time, considerably reducing the lead time of products.

(2) Reduction of investigations due to human errors and reduced re-testing of products.

(3) More accurate results (no human intervention, for example in dilutions).

(4) Use of people's expertise in tasks other than sample preparation.

(5) Reduction of waste generated by sample preparation with a saving in waste disposition.

(6) A partnership relation with Zymark that gave an opportunity to solve any situation and help improve the methods.

### Automated acetyl analysis of cellulose acetates

**Ralph Bandy**
*Eastman Chemical, Kingsport, TN, USA*

The acetyl analysis of cellulose acetates has been automated utilizing a Zymark robot for sample preparation and potentiometric titration to detect and quantify the acetate ion formed by saponification of the ester samples. The procedure for the acetyl analysis of cellulose acetates involves solvation of the ester sample in a suitable solvent followed by hydrolysis of the acetyl groups using a strong base. Any residual base is titrated to an initial end-point, followed by titration of the acetate ion at the second end-point. The manual potentiometric procedure is both time consuming and very technique dependent, making an excellent candidate for automation. The system incorporates a Visual Basic Graphical User Interface (VB-GUI) developed in-house to serve as an interface between the analyst and the robot system. The VB-GUI also serves as an interface between the analyst and the laboratory LIMS system allowing automated data transfer.

### Automated immersion testing

**Alex W. Gutierrez**
*US Army Research Laboratory, Weapons and Materials Research Directorate, Aberdeen Proving Ground, MD, USA*

An intelligent robotic work cell has been developed to perform immersion testing of polymer composite test specimens. Immersion testing involves measuring weight changes in the composite specimens as a function of the time that they are immersed in water at a particular temperature. The robot automatically handles test specimens, operates equipment, and measures the amount of water absorbed by each specimen. Traditionally, immersion testing is a labour intensive procedure that involves a repetitive sequence of operations such as transferring liquids; blotting and weighing specimens; measuring specimen thickness and recording data. Through automation, the robot can handle many test specimens and immersion testing can be done 24 hours per day, and seven days per week. This not only improves the accuracy and reproducibility of results, but can facilitate the production of an enormous amount of data. This quantitative information is required to assess environmental durability and to guide the specification, design and manufacture of composite materials.

### Automated titration system for the assay of absorbent gel materials in diapers

**Kyoko IdA, Shunji Ishigam and Dean L. DuVal**
*Procter & Gamble Far East Inc., Kobe, Japan*

The present manual method to quantitatively determine Absorbent Gel Materials (AGM) in diapers (nappies) involves an extraction procedure followed by an acid-base titration. However, this method is laborious and time-consuming. It requires six hours for two people to analyse 40 diapers. To overcome this situation, Procter & Gamble Far East has introduced a fully automated titration system by integrating a robot, automated extraction baths and an autotitrator. The novel system contains a Zymate XP robot, three sample racks (36 pads/rack), a Mettler titrator, three bubbling extraction baths, pumps, balances and a waste container.

A paddle mixer was previously used to facilitate extraction of the AGM out of diapers. However, this method requires skilled personnel and a fairly long sample-preparation time. A new bubbling air method was developed and the results give more than 98% recovery after 6 min bubbling. Three bubbling extraction baths are used simultaneously, providing a greater number of sample preparations per unit of time.

This robotic system was developed to automatically prepare samples, react AGM with excess HCl, and titrate the excess HCl with NaOH. The amount of AGM in the diaper is automatically calculated.

### Use of a Zymark robotic system for acid-digestion with microwave technology

**Norbert E. W. Mueller and Bernhard Ciommer**
*Hoechst AG, Frankfurt, Germany*

The digestion of mineral and organic samples is the most important preparation step for element specific analysis by means of AAS- and ICP-techniques. It forms the essential basis for reliable results; errors in this stage of analysis cannot be corrected later. So these marginal requirements initiate the development of a universal digestion robot to get qualitative high grade reproducible digestions of organic substances for the atomspectrometric measurement.

The automation should certainly not be realized at the expense of flexible handling of samples and digestion conditions. Many pilot tests had to be made for combining test sample series and single individual samples. The final result was a flexible automated system, which is able to do quite varied samples series but also a single sample in one digestion cycle. The system was designed in co-operation with Zymark and Maassen/Berghof (specialists in microwave techniques).

### Strategies for integration of combinatorial chemistry synthesis and screening

**Richard Kris**
*Selectide/HMR, Tucson, AZ, USA*

Combinatorial chemistry has grown to become an important part of the biotechnology and pharmaceutical industries. Combi-chem raises some specific concerns for high-throughput screening programmes. One is flexibility. As unique libraries are produced each week or each month, for some only one project will be screened in detail (with multiple concentrations tested for each compound) while for others several selected projects will be screened at single concentrations. Hence the author's application of high throughput combi-chem screening has implemented a fairly large integrated system equipped with the hardware necessary for running assays for all current projects. Another concern is integration of screening with synthesis. Selectide/HMR is complementary microplate-based Zymate systems for synthesis and screening. Selectide/HMR's experience in automation and integration of combinatorial screening and synthesis was the focus of this presentation and of the next presentation by Stephen Felder.

### Strategies for automated microplate synthesis of combinatorial libraries

**Stephen Felder**
*Selectide/HMR, Tucson, AZ, USA*

The presentation reported on a system for synthesis of Combi-Chem libraries in microplates. Libraries are produced either as large collections of individual compounds—useful for profiling and optimization of an existing lead compound, or as a large collection of mixtures—to help search for novel chemical leads. The system uses a work-station approach. One station is for down-stream processing of synthesized compounds-washing, deprotection, either cleavage from resin or extraction of product, and preparation of the compounds for use. The other station is a multi-functional work-station for synthesis. Each function is run as a batch process for all of the microplates according to library design. Use of this approach for production of Non-Iterative Deconvolution Libraries was presented.

### Implementation of an automated preparatory purification verification system

**Michael Routburg, Rolf Swenson, Jill Hochlowski, Philip Serole, Bob Schmitt, Gene Maslana, Al Washington, Boris Minin, Sandra Mueller and Ken Matuszak**
*Abbott Laboratories, Abbott Park, IL, USA*

Abbott Laboratories is involved in creating single compound libraries by parallel synthesis. It has been found that the samples often lack the desired purity. Therefore, a purification process is sometimes required. This presentation focused on Abbott's approach to the automation of the purification of parallel synthesized compounds, and the integration of that process with the mass spectrum verification of compounds, which have been prescreened through an analytical HPLC and subdivided by purity and by quantity. The development and implementation process of the system were discussed, along with the methodology, selection criteria, direction of the automation, what worked and what did not, what was suitable and what was not.

### Efficient high throughput quantitative analysis in Glaxo Wellcome's Division of Bioanalysis and Drug Metabolism

**Julie Tomlinson**
*Glaxo Wellcome, Research Triangle Park, NC, USA*

During Glaxo-Wellcome's post-merger integration efforts, a project was undertaken to examine efficiency in the international division of Bioanalysis and Drug Metabolism. That project and its team were called High Throughput Quantitative Analysis (HTQA). The division of Bioanalysis and Drug Metabolism is made up of departments that perform analytical support for discovery, ADME and clinical, plus a strategic technology group and a systems support group. A 14-member team of scientists was formed with UK and US representatives from each of those five departments. The HTQA team collaborated to perform an evaluation, decide on solutions and make short-term and long-term implementation recommendations to management. They evaluated automated instruments and procedures, detection systems and work-flow across two US and three UK sites. Their recommendations—which were unanimous—took into account similarities and differences in HTQA needs between the sites and applications.

### Automated HPLC methods for the analysis of Zaleplon and metabolites in human plasma and urine

**D. M. Rushworth, R. Unczowsky, P. Amorusi and S. Tse**
*Wyeth-Ayerst Research, Pearl River, NY, USA*

Zaleplon is a non-benzodiazepine sedatime/hypnotic agent currently in clinical development. The analysis for Zaleplon and the des-ethyl metabolite in human plasma and urine was conducted using a high performance liquid chromatography (HPLC) method. The assay was initially validated using a manual extraction procedure which was tedious and labour intensive. Since Zaleplon and the des-ethyl metabolite are stable in human plasma and urine, this assay was an ideal candidate for automation. The HPLC method for the analysis of Zaleplon and the des-ethyl metabolite in plasma and urine was validated with automatic sample processing using a Zymate II Plus robot. In this method, sample aliquots were loaded into refrigerated racks on the robotic system. The Zymate robot was configured with the following modules: Dilute and Dissolve; Centrifuge; Test Tube Dispenser, TurboVap Evaporator and Reodyne Injector. The analytes were separated with a Beckman LC-8 column and monitored by fluorescence (460 nm). This method has been successfully validated over a linear range of 0·5–100 nm/ml in human plasma and 2–2500 ng/ml in human urine with coefficients of variant < 10% and bias < ±15%. The validation results of the robotic extraction compared favourably to the validation results of the manual method. The advantages of using the Zymate robotic system in bioanalysis include uniformity of sample processing and treatment, minimizing analyst contact with biological and chemical hazards and improvement in laboratory productivity by freeing up the analyst's time from tedious and repetitive sample preparation steps.

### Enhancing productivity for bioanalytical assays: utilization of automated SPE isolation and LC/MS/MS for steroid analysis

**Timothy Getek, Walter Mei and Shankar Hariharan**
*Forest Labs, Inc., Farmingdale, NY, USA*

Advanced state-of-the-art analytical instrumentation, such as LC/MS/MS, has allowed the analyst to run a large number of samples (100 to 300) in one day by utilizing shorter chromatographic times. In order to keep up with the pace of the analysis speed, a rapid procedure of extracting the analyte from biomatrices was needed; this meant automating the process by which samples were prepared. In this presentation, a case study of the analysis of several steroids from biomatrices was discussed. The analytical protocols were rapidly developed (typically, less than three days) at levels of 150 pg/ml in plasma. The combination of an automated SPE system and LC/MS/MS permitted the rapid analysis of a large number of samples in a few days. Rapid throughput of the data was complemented using a computer network which allowed for speedy data processing via commercially available database software.

### The RapidTrace in the contact bioanalytical laboratory: impact on data quality and sample throughput

**P. Zavitsanos and Joe Palantra**
*Biovail Corporation International, Toronto, Ontario, Canada*

Acyclovir is an antiviral compound currently under analysis at Biovail Corporation International. The assay is analysed by HPLC-UV with a Level of Quantitation of 5 nanograms per millilitre. Extensive data for the manual extraction of this compound exists in the laboratory and serves as a good baseline for comparison to the newly implemented automated technique, solid phase extraction using the RapidTrace Workstation. The presentation compared the precision and accuracy data of manual to automated techniques, as well as average comparative time lines per batch. Impact on sample throughput of the automated system was examined, in addition to projection of future sample throughput improvements.

### Automated ion exchange extraction for quantification of a synthetic amino-acid drug candidate by LC-MS-MS

**Christopher Payne, Guy Carter, Gary Whiffin and Richard Lachno**
*Lilly Research Centre, Surrey, UK*

LY35470 monohydrate is a synthetic amino acid analogue of glutamate that is being investigated for utility in various CNS disorders through activation of mGluR2 and mGluR3 receptor subtypes. An LC-MS-MS assay has been developed to quantify levels of Ly354740 in plasma from human volunteers. Deproteinized plasma extracts were purified by automated strong anion exchange chromatography using a Zymark RapidTrace solid phase extraction workstation. The positive fluid displacement design of this apparatus, where eluent delivery is controlled by syringe pump, allows the low flow rates necessary for efficient ion exchange. Batches of up to 150 samples have been processed in approximately 4 h. Further purification of samples is performed chromatographically by an on-line column switching system prior to quantification of LY35470 using a Sciex AP III+ with heated nebulizer interface. The assay is extremely robust in operation, as well as being accurate and precise. To date over 1000 clinical samples have been analysed in the range of 2–100 ng/ml.

### High throughput solid phase extraction for bioanalytical applications

**George Hutchinson**
*Anachem Ltd, Luton, UK*

**Christopher Fraudeau**
*Gilson Medical Electronics, France*

**and Marisue Paulus**
*Gilson Inc., Middleton, WI, USA*

The power and reliability of solid phase extraction (SPE) is now well proven and this sample preparation technique is widely used for a variety of compounds in an extensive range of biological matrices. As modern analytical techniques, such as LC/MS, enable shorter run times the throughput of the sample preparation becomes an important goal in method optimization. This presentation described a novel approach to the automation of conventional solid phase extraction cartridges using new hardware. By taking a minimal list approach to method design, optimization of chemistry, correction selection of sorbent mass and identification of critical extraction parameters, a throughput of more than 50 biological samples per hour can be achieved.

### Automation of extraction procedures for Zaleplon and various metabolites in biological fluids using the ASTED (Automated Sequential Trace Enrichment of Dialysates Sample Processor) coupled to HPLC

**M. Milisci, K. Tantillo, P. Amorusi and S. Tse**
*Wyeth-Ayerst Research, Pearl River, NY, USA*

The Gilson ASTED system employs a dialysis technique to separate low molecular weight analytes from complex biological matrices such as plasma, urine, and blood. The dialysis membrane used on the ASTED has a molecular weight cut-off of 15 000. High molecular weight compounds, such as proteins, that normally interfere with the HPLC separation and quantitation of analytes are excluded from the 'filtered' recipient side of the membrane containing the dialysates. Since this one-line deproteinization process dilutes the dialysates, a trace enrichment column (TEC) is used to concentrate the analytes of interest just before injection. The TEC is typically a small column containing a suitable packing material that has a high affinity for the compounds of interest. The HPLC mobile phase is then used to selectively elute the retained analytes from the TEC onto an analytical HPLC column. The ASTED system has been used as an automated on-line sample preparation procedure for biological samples that normally require tedious extraction, concentration, and clean-up procedures before HPLC injection. This system has been validated and used for the routine analysis of Zaleplon (a no-benzodiazepine sedative/hypnotic agent) and several metabolites in human plasma and urine. The linear concentration range for Zaleplon and the desethyl metabolite in plasma and urine was 0·5 to 100 ng/ml. The concentration range for the M1 and M2 metabolites in plasma was 25 to 5000 ng/ml. Inter-day precision (SD) and accuracy (%bias) were < 10% and < 16%, respectively, for all analytes.

### Extraction of broad range compounds using solid phase technology

**Margaret Raisglid and Michael F. Burke**
*University of Arizona, Tucson, AZ, USA*

Solid phase extraction has proven to be an effective technique for isolating and concentrating a broad range of compounds from a variety of matrices. one of the challenges has been selecting sorbents and solvents that are suitable to extract a broad range of compounds from a single sample. Although it is possible to select a phase and conditions that will optimize overall recoveries for a given type of sample, the recoveries of some of the individual compounds may be compromised. For example, a sample may contain analytes that range from small polar molecules to large hydrophobic molecules. If a short chain modified silica, such as C2, is chosen as the bonded phase, the large compounds can be retained, and subsequently eluted, but the small, polar compounds will pass through the extraction bed unretained. Alternatively, a long chain modified silica, such as C18, may be used for the extraction. Although the full range of compounds may be retained, it is often difficult to elute the large, hydrophobic compounds. An alternative to using an extraction cartridge containing a single bonded phase is to layer different sorbents in a single cartridge. The remaining challenge is to characterize the sorbent and solvents using stacked columns and then optimize the layered column for use with automation. Examples using several unique combinations, which are compatible with the AutoTrace unit, were presented. Layered columns have proved to be an effective approach for optimizing recoveries for broad range analytes.

### *In vitro* drug interaction studies in drug discovery—higher throughput methods

**Diane Johnson, John Janiszewski and Donald Tweedle**
*Pfizer Inc., Groton, CT, USA*

Drug metabolism studies are providing key information in drug discovery to assist in the selection of new drug candidates. The challenges of analysing large numbers of compounds in a timely manner have necessitated new approaches. Technologies used for high throughput pharmacology screens are being applied to conduct metabolism studies. Initial metabolism screens have been estabished to conduct *in vitro* drug interaction studies. These studies can be used to predict the potential for *in vivo* drug–drug interactions and can also indicate which individual enzymes may be involved in the metabolism of discovery compounds. These inhibition studies generate IC50 values (drug concentration to inhibit enzyme activity in the absence of inhibitor by 50%). Routine assays have been established in 96 well plates to measure inhibition of the major isoforms of cytochrome P450, the family of enzymes primarily responsible for the metabolism of drugs. Conducting these assays has presented problems related to enzyme lability, sensitivity to organic solvent concentration, and timely analysis of large numbers of samples. Examples of the approaches being taken and the data generated were provided.

### Benchmate assisted-chlorophenoxy acid herbicides urinalysis method for biological monitoring of exposed field applicators

**K. K. Brown, A. W. Teass, L. E. Stettler and C. J. Hines**
*U.S. National Institute for Occupation Safety and Health, Cincinnati, OH, USA*

A biological monitoring method was developed for the determination of the chlorophenoxy acid herbicides, 2, 4-dichloro-t-methoxybenzoic acid [dicamba], 3-(2,4-dichlorophenoxy)propanoic acid [dichlorprop], 2,4-dichlorophenoxyacetic acid [24D], 3-(2,4,5-trichlorophenoxy)propanoic acid [245TP], (2,4,5-trichlorophenoxy) acetic acid [245T], and 4-(2,4-dichlorophenoxy)butanoic acid [24DB] in urine. Chlorophenoxy acid herbicides are difficult to analyse by broad multi-residue analytical methods because of their high polarity and ionic properties. These compounds required derivatization for GC analysis, and the derivatizing reagent varies among published methods. This present method derivatized using pentafluorobenzyl bromide [PEB]. Unique to the method was the use of solid phase extraction [SPE] that made it amenable to automation. BenchmateTM automation allowed for overnight unattended sample preparation assistance with computer file documentation of each step. An internal standard [IS], the PFB ester, of 2,4-dichlorophenyl acetic acid [24DP], was used for quantification and quality control, and an internal instrument performance standard [IIPS], dinoseb methyl ether was used to monitor instrument performance.

Sample preparation was assisted using a BenchmateTM robotic workstation. Samples were acidifed with phosphoric acid and fortified with internal standard, 24DP, each at a concentration of 100 ng/ml. The calibration urines were blank urine samples fortified with herbicide standards. The herbicides and internal standard were extracted from the urine samples using C18 SPE cartridges, and eluted with acetone into tubes containing pentafluorobenzyl bromide and cesium carbonate. After 10 minutes of reaction time, the solutions were transferred to tubes containing hordenine that quenched the reaction, by consuming the excess pentafluorobenzyl bromide. Dilute sulphuric acid was added to the quenched reaction mixture, and the 35% aqueous solution was passed through a second C18 SPE cartridge. the pentafluorobenzyle esters were retained on the columns, and were eluted off with toluene. 2-(1-Methylpropyl)-4,6-dinitrophenol (dinoseb) methyl ether was added to each final extract as an internal instrument performance check. The extracts were analysed by capillary GC with electron capture detection.

Urine samples were analysed in batches of 30 7·0 ml samples consisting of 16 field urine samples, eight calibration urines, three quality control urines, a blank urine, and a water blank. Quality control was maintained for each sample by monitoring the peak height response (mV/ng) of the internal standard and the internal instrument performance standard. Shewhart's control charts of the dinoseb response and chromatographic efficiency (N, theoretical plates) monitored the performance of the instrument for each sample, and detected instrument failure. Control charts of the internal standard response monitored the performance of sample preparation for each sample. If the internal standard response was out of control, the robotic data audit trail allowed for diagnosis at the sample preparation step. Traditional batch quality control was also obtained by monitoring the response of quality control samples, replicates, and water blanks. Thus, the quality of each data was assured. Data processing and record keeping was facilitated by the direct linkage of Benchmate and chromatographic data files to a preprogrammed spreadsheet, which were stored on optimal disks.

The present method produced a linear detector response from 1 to 1000 ng/ml. Percentage relative standard deviation for 24D averaged 7% above the LOD. Batch limits of detection for dicamba, dichlorprop, 24D, 245TP, 245T, and 24DB averaged 34, 6, 9, 24, 19, and 18 ng/ml, respectively. Thus far, field samples ranged in 2,4-D concentration from the LOD to 326 ng/ml.

### Automation in the oligonucleotide synthesis lab—the workstation approach

**Burt Goodman, Joel Boymel, Cheryl Johnson, Brian Governski, Julie Ross-Kramer, David Van Ausdall, Ted Jones and William S. Marshall**
*Amgen Inc., Boulder, CO, USA*

The DNA Technology Group at Amgen-Boulder is responsible for the synthesis of all oligonucleotides for Amgen Inc. The process of automated DNA synthesis was approached using a 'workstation' rather than a 'system' approach. Using this approach, and integrating the use of commercially available DNA synthesizers, flexible workstations were developed that perform the following functions: vial sorting and decapping, solid phase extraction, and liquid transfer. The entire process is tracked by a database system developed in-house. The process of oligonucleotide synthesis, as well as the function and performance of these workstations, was examined.

### The development of an automated high throughput screening system

**D. Harding**
*Thurnall plc, Manchester, UK*

The automation of high throughput screening is rapidly gaining acceptance in the drug discovery world. This paper described how one such facility was developed, the flexibility of the final system, and the design process required to produce an 'industrial' level of reliability and robustness. The facility was developed for Glaxo Wellcome's Medicines Research Centre in the UK. It consists of two robot systems, one handling isotopic assays, the other non-isotopic work. The facility is designed to handle both 96 and 384 microtitre plates. Standard instruments are used, with the exception of incubators and the bulk reagent dispenser where instruments were developed especially for the application. The two systems are controlled by Thurnall's SPRINT software system—a Windows$^{TM}$ based scheduling system providing significant user flexibility, while remaining user friendly. The facility was developed to meet specific customer requirements within a short timescale. The project lifecycle was described to show how the initial requirements were converted into a working system.

### The use of reactive fragment structure transformations for automated structure generation in automated combinatorial chemistry with applications to diversity analysis

**John Cargill**
*Ontogen Corporation, Carlsbad, CA, USA*

Computer programs for the automated generation of molecular structures are required to handle the volume of data produced by recent advances in combinatorial chemistry. Typically a list of reagents and a reaction template are provided as input to these programs and a set of structures is generated as output. One demonstrated approach to this problem is based upon a parent-substituent model. In the Ontogen approach, the structure of each reagent is specified to the graph structure of the reactive fragment that is incorporated into the desired structure post-synthesis. By specifying the connection points among the fragments, the final structure can be generated without the limitation of an invariant parent fragment. The reactive fragment approach is universally applicable for the many reactions in which a parent-substituent approach fails. In addition, the application demonstrates a method of diversity analysis utilizing reactive fragments which has several desirable features.

### A robotic system for agar diffusion testing

**Morten Heide**
*Novo Nordisk A/S, Novo Alle, DK 2880 Bagsvaerd, Denmark*

**Jan Madsen and Nils B. Jensen**
*Novo Nordisk Engineering A/S, Krogshoejvej 55 DK 2880 Bagsvaerd, Denmark*

A robotic system for screening for anti-microbial activities against agar imbedded target micro-organisms has been developed and implemented at Novo Nordisk A/S. The system can carry out high throughput screening of extract collections for anti-microbial activity against up to 40 different target organisms per run. An Adept One robot is used for moving the sample racks and the target plates between the two storage hotels and the working platforms. On each target plate a 9 × 9 array of holes is punched by the robot using a custom designed tool. To avoid carry over between plates with different target organisms, the tool is cleaned in a solvent between the plates. The solvent and the surplus agar from the plates are removed using vacuum and collected in a freeze trap. The samples are distributed to the target plates by the robot using a high performance pipetting system with nine individual syringes. The pipetting tool is equipped with needles for penetrating the septa on the sample vials. To avoid carry over, the pipetting tool is cleaned in a solvent between samples.

After incubation the clearing zones are scored with regard to size, shape and turbidity using a vision system. The images and scores are transferred to a database. To protect the targets against foreign organisms, the system is enclosed in a hood with a sterile laminar airflow.

### Eight steps for non-programmers to write a Windows-based instrument controller

**Michael J. Shea**
*Recombinant BioCatalysis Inc., Sharon Hill, PA, USA*

To unite an instrument controller with a Windows interface it is not necessary to be an expert programmer. With the aid of Visual Basic, and other design tools and techniques, instrument control programs can be written with little programming background. An eight-step approach for writing control programs was described. A terminal program that can help in understanding the instrument was discussed. This also includes codes that can be reused for the implementation of new instrument control programs. This presentation described the complete creation of an instrument control program for an SLT Spectra Shell Reader instrument.

### Automated high throughput mass spectral analysis of oligonucleotides

**David Van Ausdall and William S. Marshall**
*Amgen Inc., Boulder, CO, USA*

The DNA Technology Group at Amgen, Inc. currently produces an average of 110 synthetic oligomers per day, all requiring quality assurance analysis. The addition of Delayed Extraction (DE) technology to Matrix Assisted Laser Desorption Ionization-Time of Flight (MALDI-

TOF) mass spectrometry enables the rapid mass analysis of multiple oligonucleotide samples. A Bohdan liquid spotting Automated Work Station (AWS) has been used for precise and repeatable sample placement, and a PerSeptive Biosystems Voyager DE MALDI Mass Spectrometer (MS) provides high throughput analysis of synthetic oligonucleotides. Prior to the development of these systems, the only methods of analysis with sufficient throughput were trityl cation analysis and polyacrylamide gel electrophoresis (PAGE). Unfortunately, PAGE can only give a qualitative analysis of the product and is unable to unambiguously verify the identity of the oligomers. The DE MALDI MS is able to determine the mass of each sample within approximately 0·1% of the expected mass. This accuracy means that the identity and purity of all oligonucleotides produced can be determined with an unprecedented degree of confidence. Using this automation suite, sample preparation, spotting and analysis of 100 samples can be completed in less than 90 minutes.

### A comparison of the Waters Tablet Processing System and Zymark Table Processing Workstation II

**John R. Stanley**
*SmithKline Beecham Pharmaceuticals, Hertfordshire, UK*

The Waters Tablet Processing System (TPS) is the only all-in tablet analysis package on the market which is not a customized system and, as such, is the main competitor to the Zymark Tablet Processing Workstation II (TPW II). In both cases, the end result is the processing of tablets and capsules but these two instruments are very different beasts. The TPS consists of a Source for Automation tablet processor married to a Waters HPLC set-up. The tablet processing control software and data analysis software (Waters Millenium) are inter-linked, which, in theory, allows the operator to obtain quantitative data in one seamless process. The TPW II, on the other hand, exists as an extraction tool and can be linked to most HPLC (or UV detectors) and data analysis packages which will have to be purchased separately from the TPW II.

To compare the extraction, ease of use and robustness of these instruments, a tablet and capsule formulation were analysed. The table extraction process was optimized using a fractional factorial approach (using Design Ease software), which highlighted not only the optimal but the critical extraction parameters. Ease of use and robustness were determined by user friendliness and reliability. The advantages and disadvantages of the TPS and TPW II were discussed.

### Remote control of Zymate peripheral devices with a PC

**Steve Michalczyk, Jeff Russell, Jennifr Semanchik and Kyeong Yeo**
*Bristol-Myers Squibb, Princeton, NJ, USA*

Zymark Corporation currently offers several peripheral devices to provide controllable syringe pumps, valves, relays and other electro-mechanical functions to the robotic workcells. Although they are intended for usage with a Zymate controller, these devices are intelligent and can be controlled by an external computer provided that their non-standard interface is translated to standard RS-232 specifications. In this case, a system using non-invasive hardware and providing a graphical user interface was desired to enable the use of Zymark peripherals outside of the traditional Zymark robotic workcell. The first phase of this project entailed the design and implementation of an interface to the Zymate Master Laboratory Station (MLS). The interface comprises a hardware serial communications bridge to translate RS-232 signals to Zymate electrical specifications and a PC-based device software driver which adheres to the peripheral command protocol.

### Automated inert atomosphere synthesis using the Tecan CombiTec System

**Stefan Loren, Robert J. Schmitt, Lisa M. Frey and Thomas J. Sowin**
*Abbott Laboratories, Abbott Park, IL, USA*

A series of 48 oxygen sensitive solid phase organic reactions were carried out using the Tecan CombiTec combinatorial chemistry workstation. Despite the need for different incubation temperatures, 24 Suzuki and 24 Heck couplings were run in parallel in a single reaction block to produce a library consisting of biaryls and stilbenes. Prior to the introduction of the palladium catalyst, the inert atmosphere of each indvidual reaction vessel was measured by GC-MS. All experimental results were reported.

### Design, development and implementation of automated screening methods at Hoechst Marion Roussel Research Institute

**Brent T. Butler**
*Glaxo Wellcome Research, Research Triangle Park, NC, USA*

**Jan R. Davis, Steve Busch and Robert Singleton,**
*Hoechst Marion Roussel, Cincinnati, OH, USA*

In the past several years there has been increased external and internal pressures on the part of the pharmaceutical industry to expedite the discovery and thus the time to market of new chemical entities. This is being accomplished with the use of robotics technology to screen thousands of compounds for searching for that next blockbuster drug. In 1994, Hoechst Marion Roussel Research Institute, HMR (formerly Marion Merrell Dow), invested in an automation laboratory and robotics system for directed and profile screening. The automation laboratory that was designed employs two Beckman Biomek 1000 robots with side loaders and a Zymark cell-based robotic system that uses a Packard Multiprobe 104DT for liquid handling. These systems handle a variety of assays including cell-based reporter gene assays, ELISAs and enzyme assays. The Biomeks are also used for creating daughter plates from compound stocks. The facility has a separate cell culture lab which maintains and supplies the cells for cell-based assays. The cell culture room has a Zymark robot designed for plating cells into 96-well plates.

The automated laboratory is currently capable of screening approximately 10 000 compounds/week for directed screens and 1 500 compounds/week for profile screening. The data generated from these assays are downloaded to an Oracle data base and analysed using ActivityBase. The results are readily accessible to scientists throughout HMR

### Evaluating the feasibility of automated analytical methods for the determination of drugs in medicated animal feeds

**Mark M. Walstead, Brian Mazur, Yacoob Haroon**
*Hoffmann-La Roche Inc., Fair Lawn, NJ, USA*

**and Ed Yapchanyk**
*Zymark Corporation, Hopkinton, MA, USA*

An automated method based on the Zymark Tablet Processing Workstation (TPW II) has been developed for extracting Frenolicin-B from poultry feed. The procedure weighs ground feed samples, adds solvents, and extracts Frenolicin-B by homogenization. After automated filtration, the samples are injected and analysed using liquid chromatography.

Automation of this application reduced analyst time from 3·5 h to 30 min for 25 samples. The accuracy and precision data for this analysis were similar to those found when samples were extracted employing more traditional methodologies.

Implementation of this technique for routine extraction of Frenolicin B from Type C medicated feeds was described.

### Validation of automated methods for ReVia tablets

**J. A. Short and K. R. Lung**
*The DuPont Merck Pharmaceutical Company, Wilmington, DE, USA*

Revia® (Naltrexone Hydrochloride) is a DuPont Merck drug that has been approved in the US and Canada for the treatment of alcoholism. This drug is formulated in the form of a coated capsule-shaped tablet. Due to the high volume of release and stability testing required to support the NDA activities, automated sample preparation methods were developed for assay and content uniformity testing of ReVia®.

Initially, the automated sample preparation methods were developed on the XP-arm based Zymate Tablet Processing Workstation (Zymate-TPW). Eventually, the assay method was adapted to the BenchMate Tablet Processing Workstation (BenchMate TPW).

These automated methods have been validated and were shown to be equivalent to the manual method. The validation of the method on the Zymate TPW and cross-validation of this method with the manual method were discussed. The cross-validation between the Zymate-TPW and BenchMate TPW was also presented.

### A break-even analysis model for the evaluation of capital spending in laboratory automation

**K. R. Lung, Cheryl Umbles and John DeMay**
*The DuPont Merck Pharmaceutical Company, Wilmington, DE, USA*

A finance model often used for the justification of capital spending in laboratory robotics and automation is the Payback Model. Although this model is simple and intuitive, it is too one-dimensional and fails to capture the dynamics among initial capital costs, efficiency improvements and sample load in an automation project. In addition, the Payback Model implicitly assumes the replacement of laboratory personnel with robotic equipment. However, the authors' experience demonstrates that the replacement of personnel with equipment does not necessarily take place.

Automation certainly improves the efficiency of analytical laboratories through the use of robotic sample preparation equipment and laboratory data processing tools. Unfortunately, many of these robotic and automation tools are costly and the high initial capital investments often have to be evaluated and justified from a financial perspective.

The Break-Even Model presents an automation project as an *X-Y* graph, in which the cost of the project is plotted on the *Y*-axis and the volume of samples is plotted on the *X*-axis. Improvement in efficiency due to automation is represented by a lower slope in the cost-volume curve. At the same time, the cost-volume curve for automation is handicapped by a high initial intercept associated with up front capital investment. The break-even point is where the cost-volume curve for a non-automated project (with a higher slope and lower intercept) crosses the cost-volume curve for the automated project.

The Break-Even Model summarizes the financial assumptions of a typical, automation project and can assist R&D Management in making investment decisions that are both technically and financially sound. Representative examples of improvements in efficiency, comparing projects that used automated sample preparation versus projects that used manual sample preparation, were presented.

### Using the Biomek 2000 to automate an EIA for the quantitation of polysaccharides

**R. Charbonneau, W. Long, J. Hennessey and L. Rubinstein**
*Merck & Company, West Point, PA, USA*

A Beckman Biomek 2000 liquid handling system, utilizing a Zymark robotic arm, was used to replace a manual EIA (enzyme immune assay) procedure for the quantitation of Pneumoccocal polysaccharides. The procedure is complex, involving the simultaneous microplate analyses of eight different polysaccharide antigens and includes: serial dilution of samples, plate washing, sequential addition of reagents and optical quantitation of a colorimetric reaction. The manual procedure is tedious, time-consuming, and prone to operator error. The robotic procedure is fully automated, requiring operator interaction only for initial setup and final data download into an Excell spreadsheet for analysis. The Bioworks software allows a variable number of plates to be manipulated without program alteration. The robotic arm provides the Biomek 2000 with labware, including pipette tips and reagents. Additional reagents, including a detection reagent stored on ice, are provided via a peristaltic pump connected to a multiport valve.

### Robotic system for the analysis of semi-solids in a quality control laboratory

**M. Mercenari**
*Schering S.p.A., Milan, Italy*

Robotics can decrease analytical costs and thus increases the profit margins of drug manufacturers. Therefore Schering S.p.A. and FKV, Sorisole (BG), representative of Zymark Corporation, USA, co-operated to try to obtain an automated system for the analysis of semi-solids.

The development of the robotic system involved four steps:

*Study*: the parameters of the methods used for the analysis of semi-solids were examined to check the necessity and the frequency of the changes to be made for analysing different products. Then the feasibility of dosing the components of most of the products without human intervention was checked.

*Project*: foundations were laid for the realization of the robotic system by checking the compliance of the technical answers to the requirements in the study phase.

*Realization*: various working stations of the robotic system were built and assembled to verify the connection between the different devices. This phase was realized by FKV and ended only when every working station worked perfectly, either separately or connected with the other.

*Validation*: this began after the definitive installation in Schering's laboratory and involved checking the method's ruggedness, product by product, which was done by means of statistical calculations. This was the most delicate phase, where the practical, 'not robotic' problems occur and have to be solved, because they cause difficulties in the use of the automated system.

### Comparison of manual versus automated content uniformity analysis of pharmaceutical solid dosage forms using the Zymark TPW II

**John Kerr**
*Glaxo Wellcome, Inc., Greenville, NC, USA*

One of the most common tests requested during development of new solid dosage forms of a particular drug is content uniformity. Depending on the matrix, manual preparation time per sample can range from minutes to hours. These lengthy procedures result in inefficient use of the analyst's time and training.

This presentation demonstrated the potential time and cost savings benefits in converting lengthy manual methods to automated methods for two solid dosage forms. One product required over three hours to manually prepare one sample for content uniformity analysis. With some modification, the method was reduced to 10 min of preparation time, including time for injection onto HPLC, using the TPW II. A second product required over one hour of sample preparation. Again, with some modification, the preparation time was reduced to minutes. Comparison of the cost savings using the automated versus manual methods was discussed.

### BenchMate automates DNPH preparation of PPM aldehydes in aqueous samples

**Stephen L. Wellons *et al.***
*Union Carbide Corporation, South Charleston, WVA, USA*

A Zymark Benchmate has been used to automate the sample dilution and 2,4-dinitrophenylhydrazine (DNPH) derivations previously done manually. This labour intensive procedure took an experienced technician about 15 min per sample. The Benchmate procedure only takes about 3 min (per sample) of technician involvement and requires less expertise on the operator's part. The procedure is applicable to samples that are aqueous or miscible in water. The automated procedure was found to be equivalent to the manual one and works for concentrations of aliphatic aldehydes (glycolaldehyde, formaldehyde and acetaldehyde have been tested so far) from several hundred down to 1 ppm. The reactants are cleaned up on C-18 solid phase extractions (SPE) cartridges. The resulting solutions are analysed by liquid chromatography (LC). Fractional factorial designed experiments were used to determine the principal effects of the procedural steps. The components of variance between the Benchmate and LC portions were estimated using nested designed experiments.

### PCB determination in transformer oils sample preparation with BenchMate™ SPE-Workstation and TurboVap® SPE-Workstation

**Andrea Joeris-Viethen and Regine Weber**
*GEW Köln, Germany*

According to the PCB-Verbotsverordnung of 1989, which is now part of the Chemikaliengesetz, it is no longer permitted to operate transformers with cool and isolation oils that have more than 50 mg/kg total PCB content according to LAGA. The determination of the PCB content follows DIN 51 527. There are six PCBs specified, which are determined vicarious for the total PCB content given by LAGA (sum of six PCBs multiplied by five). The samples of transformer oils are cleaned up by liquid chromatographic preparation and the sample concentration can be automated conveniently and reliably by the BenchMate SPE Workstation and TurboVap LV Evaporator.

### Development of a fully automated flow injection system

**Michael J. Tutor and Seth Gilmore**
*VMI Research Laboratories, Lexington, VA, USA*

Flow Injection Analysis(FIA) is a very popular technique. It is inexpensive compared with batch automation methods and it requires relatively simple equipment; it is amenable to a variety of analyses, conserves reagents, reduces contact with possibly hazardous chemicals, and is often many times faster than batch methods of analysis.

The VMI Intelligent Automation Group is developing a fully automated FIA system. With the use of the LabView 4.0 graphical interface programming software, a point and click interface has been created to allow minimal training to operate the system. The system consists of a variable size syringe pump, an automatic sample injector, a digital I/O board that controls a set of solenoid valves, and a spectrophotometer. The FIA system has been used to automate the analysis of several metal ions, including Co(II), Fe(II), Pb(II), and Hg(II).

### Modification of standard methods of water analysis to simplify the adaptation to flow injection analysis

**Michael J. Tutor, Seth Gilmore and Aaron M. Hamilton**
*VMI Research Laboratories, Lexington, VA, USA*

Flow Injection Analysis(FIA) is a popular means of laboratory automation. It is fast, economical, conserves reagents, reduces contact with dangerous materials, and is also flexible in the types of analyses that can be performed. A system consists of a pump for the reagents, an injection valve to inject the analyte and a reaction coil that leads to a spectrophotometer. FIA can also accommodate in line extraction cells for hazardous or complex separations.

Standard methods that require heating or waiting periods to allow a complexing reaction, or extraction techniques involving organic reagents make the use of FIA impossible or extremely difficult. It is often much easier to adapt methods to FIA than try to accommodate a new or complex technique to the method. Methods that require extraction, heating or waiting periods to allow the complex to form can possibly be catalysed or solubilized by the use of surface active agents.

The VMI Intelligent Automation Group is developing a fully automated FIA system. Several batch methods have been modified with the use of surfactants to accommodate the use of FIA. The standard dithizone method for lead, mercury, cadmium, zinc, and copper requires a liquid–liquid extraction with a nonpolar solvent; surfactants have been used to eliminate this step. Surfactants have also been used to eliminate heating and waiting periods in the standard methods of cobalt and nickel. Such modifications have made these methods easily adapted to FIA.

### The modification of standard methods of analysis of water for automation on a dual robotic system

**Michael J. Tutor, A. P. Gehring and Aaron M. Hamilton**
*VMI Research Laboratories, Lexington, VA, USA*

One of the primary reasons that laboratories are automated is to increase the sample throughput by increasing the speed and by reducing the human error that can result in inconsistent data or the need to rerun samples. Automation also has the potential of around-the-clock operation. Many standard methods are not excessively difficult to automate, but extensive procedures can actually take longer on a robotics system than if done mutually. It is often beneficial to consider adapting certain methods to the robotic system as opposed to adapting the robot to run these methods.

The VMI Intelligent Automation Group is using an in-house colorimetric analysis program (SPECTRO) to optimize and modify standard analytical methods for use on a dual robotics system. The Spectro program is designed to be broad and universal so that several new types of analytes could simply be added to the system without reprogramming the basic procedure. Several ions (including lead, zinc, mercury, copper, cadmium, cyanide, aluminium, chromium, and cobalt) have been optimized for automation. Certain methods, such as aluminium and cobalt, underwent extensive modification to ease the adaptation of these methods to automation using the in-house program.

The Eriochrome Cyanine R method for determination of aluminium is published as a procedure that involved adding four separate reagents to the test solution to produce the desired colour for detection. This procedure was not compatible with the in-house colorimetric analysis program. It was modified so that the reagents responsible for the development of colour were combined, and the combined 'colouring solution' was then added to the test solution, and then analysed after the required time. This modified procedure is now compatible with the in-house program.

The complexing agent 4-(2-pyridilazo)resorcinol(PAR) is used in many standard colorimetric methods of analysis. Cobalt analysis with PAR requires heating and waiting periods, along with addition of masking agents after the complexing reaction has occurred. This made the automation difficult and time consuming. The method was modified using the surface active agent Triton X 100 to catalyze the reaction and eliminate the need for heating and waiting periods (Tutor M. J., *VMI Undergraduate Research Review*, vol. II, 3, 1996). These modifications made the method much easier to adapt to the in-house system.

### The design, construction and testing of a dual robotic system

**Michael C. Zirkle**
*VMI Research Laboratories, Lexington, VA, USA*

Two primary reasons for automating analytical methods in a chemistry laboratory are to save time and to decrease human interaction. Software packages are constantly being updated to include new scheduling algorithms and other programs that allow the user to do more with less. There comes a point where complex software solutions no longer make significant improvements in performance, and a new path needs to be followed. Rethinking the hardware configuration is the next logical step.

The VMI Research Laboratories Intelligent Automation Group, a multidisciplinary team composed of chemists and programmers, has combined the Zymate II and Zymate XP robotic systems, with in-house and commercial software systems, to create a dual robotic system for versatile colorimetric analysis under a Windows NT™ environment.

A description of the automated system was presented here, along with the requirements posed to the system and the software and hardware utilized.

### A novel microwave autoclave for automation of a pharmaceutical research application

**W. Lautenschlager, W. G. Engelhart and M. Metzger**
*Milestone MLS, Riviera Beach, FL, USA*

Hundreds of laboratories currently utilize microwave systems to accelerate the preparation of samples for research purposes, quality control and process control analyses. Conventional microwave systems with multicode cavities and doors are designed for manual operation.

A new microwave autoclave eliminates the impediments to automating microwave chemistry procedures. The ultra CLAVE system combines microwave heating and high pressure vessel technology allowing chemical reactions to be conducted at pressures and temperatures up to 200 bar (2 900 psig) and 350°C. The system is specifically designed for semi-automated batch processing of multiple samples. Full automation is achieved for interfacing a laboratory robot arm to load/unload sample racks.

Fundamental design and operating principles of the system were presented along with examples of pharmaceutical research applications performed in the system, synthesis reactions, solvent extractions, peptide/protein hydrolyses, acid digestion of implant materials, etc.

### Radiopharmaceutical automated dosage system

**Rodney Stockton**
*SLR Systems, Richland, WA, USA*

**James W. Pancy and Mark Nybo**

*Medi-Physics, Inc., Spring Lake, MI, USA*

Radiopharmaceuticals are used in nuclear medicine for various diagnostic and therapeutic procedures. Normally, the radiopharmaceutical is drawn into a disposable syringe by a pharmacist. Although single dosages do not present a health hazard to the patient, the preparation of 300+ dosages per day by the pharmacist presents a considerable exposure hazard. This presentation described an automated system for drawing radiopharmaceutical dosages into disposable syringes and placing them into lead shields.

This system has been in operation for several months at the Chicago facility of Medi-Physics, Inc. It is capable of drawing 60+ doses per hour. Future modifications will increase the throughput to 120+ doses per hour. The system is based upon the Mitsubishi RV-M2 articulated arm robot and custom hardware and software designed (in conjunction with Medi-Physics personnel) by SLR Systems. Manufacture of the system was performed at SLR Systems' facilities in Richland, WA.

The system is controlled via a 486 computer, programmed in Microsoft Visual Basic. Two microcontrollers (BS2 from Parallax, Inc.) are incorporated to provide intelligent digital I/O for sensors, pneumatic slides, conveyors, and turntables.

### Custom engineered automation for scientific research

**Ed Ball and Dave McCampbell**
*Midwest Research Institute, St. Louis, MO, USA*

MidWest Research Institute (MRI) has been providing contact scientific research for 50 years, in fields such as energy, environmental, health, and transportation. Automation solutions have been provided for the laboratory research scientist for almost 10 years. The automation group is a multidisciplinary group with backgrounds in mechanical engineering, engineering design, artificial intelligence, computer programming, machine vision, analytical chemistry, and biology.

Automation specialists at MRI have developed robotic systems to increase the throughput and precision of such tasks as natural pesticide screening, oncogene inhibitor screening, biological matrix extraction, and mineral content determination of food products. Some of the custom systems and modules developed by the automation group at MRI were described.

### Dimensional analysis of rigid polyurethanes with Windows '95

**Steven E. Robbins and Michael E. Rusak**
*Air Products & Chemicals Inc., Allentown, PA, USA*

Rapid polyurethanes are used primarily as insulation and subjected to a variety of environmental conditions, typically from 20° in commercial refrigerators to 160°C in ovens. The dimensional stability, or resistance to deformation, is a key material property, indicative of the material's long term behaviour in the specific application.

A Windows '95 platform has been used to automate sample manipulation, instrument control and data acquisition. The system includes several controllable sample carousel racks, dimensional measurement gauges, positional control devices, optical sensors, and a mass balance. This presentation described the operational simplicity of a complex customized system as well as some of the key building blocks used.

### Challenges and opportunities in high throughput screening: implications for new technologies

**John Major**
*ZENECA Pharmaceuticals, Macclesfield, UK*

In response to mounting competitive pressures, the current trend in the pharmaceutical industry is to shorten the time scale for all aspects of drug discovery. While advances in computation, structural chemistry and molecular modelling are facilitating rational design activities, empirical screening continues to play a crucial role in lead identification. Because the ability to test large numbers of compounds quickly and efficiently can provide a competitive advantage, high throughput screening (HTS) has become a key goal. To achieve the necessary productivity, effective integration of compound supply, assay operation and data management is essential. HTS is a high technology enterprise that must take full advantage of the latest advances in bioscience, biotechnology, engineering and electronics. There is a constant dilemma, however, in relation to when well established, mature technologies should be replaced by new methods that promise to deliver spectacular advantages. The final decision must be based on weighing up the promised benefit against the cost and risk. While huge challenges face the pharmaceutical industry, there are also opportunities for those companies that can identify and implement new technology effectively. The requirements for efficient HTS and the implications in relation to assay technology and automation were discussed.

### Transforming robotics into an infrastructure for the future

**John Babiak**
*Wyeth-Ayerst Research, Princeton, NJ, USA*

Advances in high throughput screening frequently occur by interfacing new automated peripheral devices or workstations which perform critical functions onto an existing robotic core system. In the past, these interfaced devices have included liquid handlers, incubators and detectors. As new peripherals are integrated to a core robotics system, new opportunities and higher productivity are realized. The key to successful progress in robotic-based automation is the perception and utilization of the core robotic communications and operating systems as an infrastructure upon which all workstation interfaces are developed. In practical terms it is necessary to answer three questions when interfacing a new device. (1) Can the device be made compatible with the dexterity of the robotic arm? (2) Is it possible to communicate with the device using standard protocols consistent with the existing robotic operations and scheduling architecture? (3) Are the benefits derived from interfacing the device significant? As the three questions imply, a robotic arm will only become an infrastructure for future development and success if it is part of an open architecture which supports expansion both physically and operationally.

### High throughput screening for novel lead compounds using defined biochemical assays

**Berta Strulovici and Sarkiz Daniel-Issakani**
*Tularik Inc., S. San Francisco, CA, USA*

The approach Tularik has taken to the discovery of novel lead compounds is the screening of hundreds and thousands of test materials from various sources against a diversity of biochemical assays in robotic format. This is achieved by integrating the organization of the test compound library with a relational database, the development of biochemical assays in a robotic-friendly format and high throughput screening. Particular attention is being paid to the use of automation in all aspects of the drug discovery operation.

To ensure structural diversity, Tularik has assembled a large library consisting of compounds of known structure, and natural product extracts from various sources such as microbial, plant and marine extracts. To fulfil immediate screening requirements and at the same time ensure longevity, our libraries are aliquoted in replicate 96 well plates. All compound information is electronically uploaded into a relational database.

Automated data analysis software has been created, which is based on the 4D program for Macintosh, to handle the vast amount of data generated. This is a relationally integrated custom application. The data from robotic screening is electronically uploaded, and so are the follow up results. Virtually every endpoint 96 well reader exports data in a different format. A driver was designed for each detection system to convert raw plate data to a single format for analysis. The database flags the 'hits' for quick recognition and generates comparative reports.

The targets in which Tularik is interested are novel. Therefore, a major challenge is the design of biochemically sound, valid assays, the ability to automate them and implement screening expediently. At present, several types of assays are running simultaneously in robotic format: protein kinases, a variety of enzymes involved in gene replication, protein/peptide interaction assays, and protein/DNA interaction assays. The assays are fully automated using Zymark robotic systems on which we integrate and customize all the hardware and software required by the assay flow chart. This includes the integration of one or two different detection systems such as a luminometer, fluorescence spectrophotometer on the same robotic system. Using these systems, a throughput of 1–2 million data points per year is achieved.

### Management of a centralized high throughput screening facility

**Mark D. Lister**
*Sphinx Pharmaceuticals, Durham, NC, USA*

High throughput screening programs have seen a resurgence as the preferred method for lead generation in drug discovery. Due to recent technological advances, such as liquid handling automation, assay miniaturization and imaging plate readers, the length of time to run a screen on any given target has gone from years to a few months or weeks. There are two prominent approaches to screening: a single centralized laboratory or a number of decentralized laboratories, often divided by therapeutic areas. Through its acquisition of Sphinx Pharmaceuticals, Eli Lilly and Company has established a centralized screening approach to its drug discovery program. In the past, screens were run by personnel trained to do a specific task where little or no scientific foundation was necessary. The present paradigm requires personnel with higher skill levels. These include a solid background in biology, an aptitude for automation and computers, and an ability to work in a team atmosphere. The operations group at Sphinx consists of 10 people who run, over the course of the year, 15 or more screens of all types (i.e., receptor, enzymatic, cellular, etc.). Methodologies employed range from semi- to fully-automated. Along with validating the automation and running the screen at high volume, managing screen operations involves input on technical feasibility of assay protocols of upcoming screens. Furthermore, it is necessary to continually seek out new technologies that will not only increase throughput but increase the probability of finding good drug candidates.

### The versatile TPW II—from powder to liquids

**Simon Smith**
*SmithKline Beecham Pharmaceuticals, Crawley, UK*

Following on from the success of two BenchMate Table Processing Workstations, the TPW II laboratory upgrade programme was developed and executed with the omission of any instrument downtime. The existing two systems have been replaced with two TPW II systems and a third was purchased primarily to support new product introductions.

The main justification for the purchase of the TPW II was for the analyses of powders, namely compression mixes during the quantification of the manufacturing process. The notion that liquid samples, for example viscous syrups, could be analysed using the quantitative powder handling module did not occur until after installation. The powder module was extensively tested within our laboratory environment during Zymark's beta testing programme. The experience gained here and also subsequent development validation efforts was described in this paper.

Several key automated analytical method validation routines have been developed for the TPW II encompassing all types of product formulations. This has aided the development of automated methods both within the laboratory and the R&D environment where the majority of method development and validation occurs before transfer to the Crawley New Production Introduction facility.

### Segmented automation techniques in a development environment

**Harnath Doddapaneni, Nagesh Palepu and John Jushchyshyn**
*SmithKline Beecham Pharmaceuticals, Upper Merion, PA, USA*

SmithKline Beecham Pharmaceuticals currently enjoys a large pipeline of commercializable compounds, as these compounds move to formulation selection and phase III testing the analytical efforts to produce data in a GMP manner tax the capacity of manual instrumentation. Automation presents a challenge because of the diversity of compounds, the unique requirements, and the level of training within each development team.

A flexible approach to automated dissolution was presented, which employs segmented workstations and a Zymark dissolution device of other automated dissolution samplers. Each dosage unit under development employs only those stations required for the task, each station is easy to use or program and requires little training. Stations not in use for the dosage unit under investigation are free to perform other tasks. Strategies to increase throughput and assure quality of the Zymark dissolution device were described. Data collection and automated reporting of non-routine dosage testing were discussed.

### Automated dissolution testing using the Zymark Multidose

**Joseph L. Schmitt, Ruben Lozano, Steve Conder and John M. Joseph**
*Bristol-Myers Squibb Company, New Brunswick, NJ, USA*

The Zymark MultiDose is a non-robotic dissolution workstation for the automation of USP Apparatus 2 (paddle) dissolution profiles. MultiDose can automate up to eight standard 6-vessel dissolution tests without operator assistance. Analysis can be performed using on-line UV or off-line by HPLC.

MultiDose frees up analyst's time by automating crucial steps in dissolution testing such as; media sparging (using helium), preheating and dispensing into two vessels; verification of vessel temperature; dropping of dosage forms (either in batch mode 'all at the same time' or serial mode 'one at a time'); collection and filtration of sample aliquots at prescribed times; and cleaning of vessels at the end of the test. These steps can be repeated for up to eight batches of tablets.

### Automated dissolution testing in pharmaceutical development

**Christine Hecht**
*F. Hoffmann-La Roche Ltd, Basel, Switzerland*

Requirements regarding time to market, cost effectiveness and cGMP compliance in pharmaceutical development have dramatically increased in the last few years and will continue to do so. This means, among other things, that different dosage forms, dosage strengths and packaging configurations of a new drug product are to be developed in parallel and before efficacy and dose range have been finally established.

One of the most important aspects in the characterization of a new drug product is its dissolution behaviour. Multipoint dissolution profiles are consistently required for release and stability testing throughout the whole development. Together with the increased number of samples and the time and cost constraints, this means that the automation of dissolution testing is mandatory for most analytical laboratories working in the field of pharmaceutical development.

For the above reasons, a Zymate System with the capability to perform fully automated dissolution testing of solid oral dosage forms using USP Apparatus 1 or 2 including online spectrophotometric or HPLC assay of the samples has been installed in the area of Development Products Analytics of the Pharmaceutical Quality Control and Assurance Department of F. Hoffmann-La Roche Ltd. This presentation described technical design, operational environment, validation and practical experience acquired in routine operation of the Zymate Dissolution System.

### Ten years of robotics and laboratory automation in a production control laboratory

**Julius Brown**
*Eastman Chemical Company, Columbia, SC, USA*

Carolina Eastman Division (CED) of Eastman Chemical Company produces polyethylene terephthalate (PET) at the world largest single site PET production facility located in Columbia, South Carolina. CED Polymers Laboratory operates 24 hours per day performing analytical testing of products and process samples for monitoring production processes. The laboratory analyses an average of 1800 samples per day (> 3800 results) using a variety of analytical techniques. Because most results are used by process operators in real-time to control or monitor processes, most samples are processed through the laboratory in 1 to 4 hours. In order to meet the demands for efficient, accurate, and timely testing CED has operated Zymark Robots and other laboratory automation since 1986. These systems are operated 24 hours a day seven days a week. CED has realized gains in labour efficiency, as well as reduced exposure to noxious chemicals through the use of automation. The robot systems have evolved from Zymate I to Zymate II to XP Robots with System V controllers. The robots perform sample preparations for different product analyses and are interfaced to an MIS system for automatic data reporting resulting in completely automated analyses. This presentation described robot and other automated applications, the evolutionary process for their improvements, and discussed the benefits and challenges to implementing and maintaining these systems.

### Management's view of the design, construction and installation of a custom robotic system

**V. E. Clay, P. Y. Yen and S. L. Widmer**
*Bayer Corporation, Stilwell, KS, USA*

The Environmental Fate Team at Bayer Corporation Agriculture Division is responsible for the development of pesticide residue methods and the annual analysis of thousands of soil and water samples. Unique residue methods are required for each active ingredient and its major environmental degradates. The methods must provide separate quantitation of each analyte in the part-per-billion to sub-part-per-billion range. Samples from EPA-mandated soil dissipation and ground water monitoring studies must be analysed promptly and efficiently. To meet shortened product development schedules without increasing staffing, custom robotics systems were purchased.

The conflicting needs of a high throughput for efficient sample analysis and adaptability for yet-to-be developed residue methods led to the initial design and construction of a functional, but unreliable robotic system. Using a 'Win-Win' approach, Zymark and Bayer abandoned the initial designs, costs and deadlines and redefined all goals and operating constraints. New robotic systems, which are much more efficient, flexible and reliable, have been constructed and installed. It is believed that the redesigned systems, with updates and modifications, will play an important role in the soil and water analysis method development work for many years to come.

### Versatility—the key for success of automation systems in research & development laboratories

**Ramasamy Tamilarasan, Paul L. Morabito, John J. Zieman and P. D. Hazelwood**
*The Dow Chemical Company, Midland, MI, USA*

Research and development (R&D) laboratories and Quality Control (QC) laboratory represents contrasting paradigms of operation with different modes of success for automation systems. QC laboratories typically have large numbers of similar samples to be analysed using well defined procedures, and thus high sample throughput is the major impact of automation systems. R&D laboratories commonly have various types of samples to analyse and analysis procedures often must be developed. The automated system should be simple and flexible for multiple users with several different procedures. This presentation described such a versatile system recently developed for preparing samples in an analytical R&D laboratory. The system is capable of preparing samples using seven different predefined methods and one user configurable method. User-friendly graphical interface provides easier data input and output.

### Managing automation in an environmental testing laboratory

**Lars W. Lindquist**
*WMX Technology Center, Inc., Geneva, IL, USA*

Today, more than ever, there is a need to automate processes to drive costs lower while improving efficiency. Automating projects requires careful thought and planning to obtain success. Often a project can be better addressed if a phased in approach is used in the development stage. One approach used at the WMX Technology Center over an eight year period to develop an ongoing successful automation program was described. New workstations and system enhancements were also discussed.

### The creation of an integrated data handling system for automated organic synthesis

**Chris Green**
*ZENECA Pharmaceutical, UK*

Automated chemical synthesis, of novel chemical entities for biological screening, has been carried out in the author's laboratory for four years. To undertake effective organic synthesis on a Zymate XP Robotic system requires a considerable amount of data manipulation. Originally, this was a tedious process involving file transfers to and from a VAX system. With recent PC and network advances, software packages (Excel, Word, Isisbase, Isisdraw, Project Library) can be run which enable the data to be managed effectively.

### Data management in the oligonucleotide synthesis laboratory

**Joel L. Boymel, Burt Goodman, Cheryl Johnson, Brian Governski, Juli Ross-Kramer, David Van Ausdall, Ted Jones and William S. Marshall**
*Amgen Inc., Boulder, CO, USA*

The DNA Technology Group at Amgen has the responsibility of synthesizing the oligonucleotides necessary for Amgen's research needs. In the last five years the demand for oligonucleotides has grown exponentially. From 1991 to 1994 the DNA manufacturing process changed drastically. The constant state of flux made a traditional database system too slow to respond to changing needs. As a first iteration, it was decided that a user modifiable, flat file system would be used. Over the next few years the manufacturing process settled and the data system evolved to handle the increasing load. Orders were electronically cut and pasted from e-mail and macro utilities were utilized to automate redundant processes. The data system was able to adapt quickly, but quickly became a difficult to manage conglomeration of sofware packages from many different vendors, loosely held together by macro tools that were stretched to their limits. Eventually, excessive time was spent ensuring data integrity, indicating that this system's limits had been reached.

In 1995 the next iterative change in the data management system occurred. Since a workable system was in place, an open, expandable system could be designed. Computer platforms were chosen to best fit the authors' situation. A client-server system was chosen to ensure that work could be done from any location. Touch screens and barcode scanners were used to speed the processing of oligonucleotides. Orders were placed directly into the sytem over the network using a small external interface, elimnating operator manipulation of sequences. File servers are used to transfer data to and from external robotic systems. More information is tracked, and reported, with less operator input and error.

The only thing constant is change itself. The best we can strive for is to manage that change as best we can. The modularity and connectivity of the current system will allow seamless upgrades when the need arises.

### Electronic laboratory notebooks: The R&D team computing and the needs, the systems and the consortium

**Rich Lysakowski**
*Team Science, Inc., Sudbury, MA, USA*

An intensive, multi-company study was started in October 1994, called 'R&D Team Computing Study'. R&D Team Computing Systems contain a large number of subsystems or components, including electronic records, management systems, electronic record keeping, project data and document management systems, workflow, groupware, and other types of collaboration systems. The purpose of an R&D Team Computing System is to foster better collaboration of professionals working on complex product research and development projects, in order to greatly reduce cycle times and improve the quality of both products and customer services.

The R&D Team Computing Study started with surveys of hundreds of users' needs in the pharmaceutical, chemical, biotechnology, and materials industries, and resulted in a master list of requirements for R&D team computing systems for FDA—and EPA—regulated industries where patent applications are routinely filed to obtain proprietary advantage. The next step was to profile the most popular vendors and systems in the marketplace, based on demand and priorities set by users. Finally, a multi-volume report was written that outlines the background for the business, regulatory, legal, technical, and social needs, the profiles of fit for various vendors and systems, along with summaries for various levels of management and end users.

The R&D Team Computing Study represents the culmination of several years of work to understand and specify the broad range of requirements of global, collaborative electronic notebook systems, which operate at the intersection of law, commerce, sociology, science, and technology. The results of the study pave the way for better integrated systems to be built that significantly enhance global collaboration on complex R&D projects, while addressing the needs for reliable and trustworthy record keeping and records management.

This presentation described the results from this comprehensive study including: the needs survey; vendor technology profiling project; bottom-line results; and the R&D team computing consortium.

### Increasing analytical laboratory productivity by simplifying complex processes

**Richard G. Poser**
*Dura Pharmaceuticals, San Diego, CA, USA*

Process re-engineering has become the fashion. As companies are compelled to do more with less, they have turned to the science of re-engineering in the hope of enhancing the productivity of their precious remaining resources. Research, development and production facilities have become more efficient and productive by implementing new technologies. Regulatory requirements demand additional testing in support of stability, cleaning and validation programs. These factors have created tremendous new demands on the analytical testing and control laboratories that support these operations. Lab managers with limited resources constantly face increasing workloads, shorter deadlines, and a demand for more rapid sample turnaround.

It is a common oversimplification to equate laboratory re-engineering with introducing automation. Re-engineering is about improving efficiency and productivity of a system. While re-engineering analysts frequently recommend automating a manual process, automation is only one of several solutions a skilled system analyst may utilize to improve the productivity of a system.

Re-engineering should begin with a thorough analysis of the process, paying particular attention to how work and information flow through the system. This may reveal redundant, archaic or unnecessary steps that can be simplified or eliminated. Only *after* the system is simplified as far as possible should automation then be considered, and only then if further gains are cost effective.

A case study was presented, showing how an analytical support laboratory was able to permanently increase testing capacity and improve test quality while reducing average sample turnaround from 60 to four days. This was achieved following a thorough process analysis that revealed how data flow could be simplified, delegation of sampling responsibility and sequential testing to eliminate unnecessary testing. A second case study illustrated how process analysis and simplification accelerate document review and approval prior to automation of a regulatory document routine system.

### The role of automated systems in laboratory re-engineering

**Michael L. Rutherford**
*Eli Lilly and Company, Indianapolis, IN, USA*

As more and more emphasis is being placed on improving laboratory efficiency in order to provide more analytical data at reduced costs, provide higher levels of customer service, and generate more timely results, many laboratories are forced to change how they are doing business. Re-engineering the workflow of the laboratory by reorganizing the functional group is one approach to this situation. This approach can provide better focus, dedication of resources, improved customer service, and reduced cycle times. However, this approach often does not reduce resource requirements and costs since some replication is necessary to meet the customer service requirements. For years, robotics and automated workstations have been utilized to improve staff utilization, as well as improve the quality of results. Incorporating automated systems into the workflow improvements can provide even greater benefits in many cases, whether part of the original re-engineering process, or as further enhancement to the plan. To demonstrate the benefits of incorporating automated systems in the re-engineering process, several scenarios within Eli Lilly were presented.

### Meeting a client's needs using a Zymark Multidose in a contract laboratory setting

**Timothy D. Rhines**
*BAS Analytics, West Lafayette, IN, USA*

BAS Analytics, a contract laboratory for the pharmaceutical industry, was challenged by a client to perform a large scale stability program. Each method required two sample pulls and two dilutions before injection into an HPLC. At the time the project was presented, BAS Analytics had two manual dissolution units. The program required potency, dissolution, TLC impurity screening, tablet hardness, tablet thickness, and moisture. Through the multiples of tablet potencies and packagings a total of 96 samples were set on stability. The task facing BAS Analytics was how to complete the required dissolution work for the three-month stability time point within a three week testing window. This stability protocol requires two dissolution methods to be performed. Previously, most of the work was done manually. BAS Analytics identified that automation was the best means for growth, without adding excessive staff. Zymark was contacted in late April of 1995 and an order was placed with them for a MultiDose Automated Dissolution Workstation, and a BenchMate II. Instrument performance validation of the systems was performed in June, as was the automated method validation. BAS Analytics was ready to test the samples when they arrived. The co-operative effort between Zymark, the client, and BAS Analytics resulted in data delivered on time for submission. The validation process, time schedule, issues and benefits experienced at BAS Analytics were discussed.

### Rosie the robot. Laboratory automation during the war years, 1941 to 1945

**Kevin Olsen**
*Wyeth-Ayerst/Lederle Labs, Pearl River, NY, USA*

In our own day, managers often cite the gains in productivity as the primary reason to automate laboratory operations. This is hardly new. During the Second World War, shortages of skilled labour and materials were felt in the chemistry laboratory. Doing more with less was not a matter of corporate policy, it was a matter of national survival.

An amazing variety of automated devices were created between 1941 and 1945. Some were designed to save labour, such as the automated distillation units seen in the petroleum industry or other organic chemistry laboratories. Certain automatic titrators, polographs, recording instruments and water still also fall into this category. Other equipment was intended to conserve strategic materials, like an all-glass constant-rate reagent addition device, specialized control relays and a constant temperature bath that was made out of a lamp globe, thermostat, and heating device, all for less than four dollars. Still others improved assay performance by automating steps that were prone to human error.

Although the technology has changed, the reasons to automate have not. These devices were largely constructed by end users who were working alone. This fact illustrates something else that has not changed, the most important ingredient in automation is not the hardware, it is the imagination.

### Solid phase extraction of antidepressants from mouse, rat and human plasma utilizing robotic workstations

**Patrick J. Faustiano, Christopher D. Ellison and Karl P. Flora**
*U.S. Food & Drug Administration, Laurel, MD, USA*

Antidepressants are used clinically to treat mental illness. Their widespread use and the large range of dosed utilized for the treatment of depression have currently revealed and expanded need for monitoring of plasma drug levels because of inter-individual differences in both steady state plasma levels and metabolism. Drug monitoring of patients undergoing long-term treatment with antidepressants may provide a more accurate determinatoin of an individual's appropriate therapeutic dose. Recent FDA studies required monitoring antidepressant plasma drug levels in rodents for pharmacokinetics scaling to humans. Automated methods that are reproducible and accurate have proved essential for the preparation of large numbers of pre-clinical and clinical samples.

The authors have developed and evaluated automated solid phase extraction methods for two antidepressant compounds and their pharmacological active metabolites from plasma: a first generation tricyclic antidepressant, amitriptyline, and a second generation selective from mouse, rat and human plasma using BenchMate robotic workstations. Plasma drug levels, pharmacokinetics and metabolism of relevant clinical doses from native and chronically treated animals were determined by high pressure liquid chromatography with ultra-violet detection. Robotic workstations now occupy a central role in the rapid development of automated methods for the preparation of bioanalytical samples.

### Microlute a semi-automated solid phase extraction system in the 96 well format

**R. A. Biddlecombe and S. Pleasance**
*Glaxo Wellcome, Beckenham, UK*

The use of liquid chromatography tandem mass spectrometry (LC-MS-MS) in quantitative bioanalysis, with analysis times typically less than 4 min, has made sample preparation the rate determining step, particularly when using solid phase extraction (SPE). The use of SPE in the 96 well format, coupled with a Packard Multiprobe robotic sample processor (RSP), allows rapid processing of samples in the batch mode. A modified vacuum manifold positioned on the deck of the Multiprobe is used to control the flow rate of the liquids through the block. The Multiprobe is used to prime, load, wash and elute the blocks. After each step, a post run customized program activates the vacuum by switching a valve. The RSP aspirates sample, buffer and internal standard sequentially and adds them to the block, the dispensing speed giving adequate mixing. The placement of the collecting plate in the vacuum manifold, prior to the elution step is the only manual intervention required. A modified HPLC autosampler (Gilson 233) is used to inject samples directly from the collection plates. The autosampler has a capacity of six blocks which even with a 2 min run time allows around the clock operation of PE Sciex API-III mass spectrometer.

This approach offers many advantages, speed (96 samples in approximately 50 min), no manual liquid handling, no vial capping or labelling and true high throughput quantitative mass spectrometry (sequential injection of up to 576 samples). The format has also proved amenable to different packing materials and packing volumes. A strategy for the complete automation of the system using a Zymark robot was also outlined.

### Design and evaluation of an automated solid-phase extraction method-development system for use with biological fluids

**Tresavon D. Parker III, D. Scott Wright and David T. Rossi**
*Parke-Davis Pharmaceutical Research, Ann Arbor, MI, USA*

An automated solid-phase extraction method development system, utilizing a Zymate XP robot and a custom-designed solid-phase extraction (SPE) manifold, has been developed and validated. This system spikes blank liquid matrix, such as plasma serum or urine, with solutions containing drug, internal standard, and up to two metabolites. Samples are then buffered or diluted with an appropriate reagent. After these samples and corresponding blanks have been prepared, solid-phase cartridges containing selected sorbents are automatically conditioned. Samples are robotically vortexed and transferred to the conditioned cartridges, and analytes are extracted. Validation of this robotic system demonstrated acceptable precision and accuracy for two types of liquid transfer including metering pump ($<$ 6%RSD and RE for $>$ 2·0 ml dispensation), syringe-based laboratory station ($<$ 2·9%RSD and 0·5%RE for volumes between 0·25 and 1·00 ml and syringe hands ($<$ 1%RSD and RE for volumes between 0·25 and 1·00ml). For two model compounds (CI-988 and PD 135158), the system effectively distinguished good solid-phase sorbents from marginal ones through precision, recovery and chromatographic selectivity. Solid-phase extraction of these compounds from human plasma gave precision (2% to 4%RSD) and extraction efficiency ($96 \pm 6\%$) comparable to results obtained from manual extractions ($92 \pm 11\%$).

### Use of the Rapid Trace$^{TM}$ SPE workstation for accelerated methods development

**Xialoi Ren and Allan Witkowski**
*BAS Analytics, West Lafayette, IN, USA*

Due to its high throughput and ability to process samples unattended, automated solid phase extraction (SPE) should enable accelerated methods development for analytes in biological matrices. To explore this possibility further, a generalized approach to SPE methods development was designed and implemented using the Rapid Trace Workstation. The model beings with pre-development groundwork, leading to an initial extraction method. The methods development phase is broken down into two portions, extraction optimization and time reduction. The automated variation of key variables (such as sorbent material and bed size, solvents, pH, volumes, speed, etc.) allows for a comprehensive range of parameters to be quickly evaluated. Finally, the optimized method is used to perform a full method validation. This experimental approach is illustrated through the development of an automated SPE assay for proprietary drug in human serum, focusing on the rational behind the methodology, the impact on assay ruggedness, and time savings achieved.

### Managing compound library production through modular automated workstation-based system

**James B. Campbell and Richard A. Wildonger**
*Zeneca Pharmaceuticals, Inc., Wilmington, DE, USA*

The advent of combinatorial libraries has infused the pharmaceutical industry with a novel set of tools to expedite drug discovery. Thus, parallel methods of synthesis and combinatorial, or mixture synthesis, coupled with high-throughput screening have greatly enhanced capabilities in identifying novel drug candidates of higher quality, and with greater rapidity, than ever before. Automation is central to the efficient implementation of compound library synthesis. The authors described some of their initiatives to develop compound library production systems for managing the numerous processes involved in high through-put chemical synthesis. Single and multi-tasking automated modules can systematize and increase the efficiency of the various processes which provide the framework of chemical library synthesis system. Thus, modules used for reagent preparation, barcode labelling of reagent and reaction containers, reagent transfer modules, off-line incubation stations and post-reaction processing stations can all be integrated to define a library synthesis system. Progress on implementation of such synthesis systems dedicated to library production was reviewed.

### Developing a high speed synthesis paradigm—the workstation approach

**Burt Goodman, Kirsten Bjergarde, Joel Boymel, Ted Jone and Larry Melvin**
*Amgen Inc., Boulder, CO, USA*

The New Leads Discovery department at Amgen-Boulder is responsible for the synthesis of libraries of compounds in an automated fashion. After evaluating commercially available synthesis automation, instrumentation was developed using a 'workstation' rather than a 'system' approach. The key to this strategy is a modular reaction block design that is portable between instruments. Reaction block modules insure that valuable instrument time is not tied up during reaction incubation periods. Using this approach, several flexible workstations which perform specific functions have been developed. One workstation performs resin loading into reaction vessels and also preparation of diversity elements in a standard format. Another workstation is devoted to transferring solvents and diversity elements into reaction vessels. A third is used for solvent washing as well as cleavage of compounds from solid supports. A separate heat/shake/cooling incubator is used for reaction incubation. To complement these systems, off-the-shelf components such as TurboVaps and speedvacs have been modified to support downstream sample processing. The function and performance of these workstations was examined.

### ACID for the Tecan: an automated combinatorial interface demonstration

**Robert M. Kennedy**
*Parke-Davis Pharmaceutical Research, Ann Arbor, MI, USA*

Automated liquid handling workstations for performing combinatorial organic synthesis are recent additions to the organic laboratory. Many of these robots are modifications of those used for biological work. Much of the recent literature describing these newly developed machines has focused on hardware.

Described in this presentation was a software suite, ACID, which was developed for the Tecan. This software arose from a need to simplify a typical organic chemist's interaction with the liquid handling robots performing parallel synthesis. Prior to this, much of the available software had been designed for biological work in which most samples are treated identically. It is the definition of combinatorial work that all samples are treated differently, hence a new approach was needed.

The 20 programs written for ACID consist of four basic groups: Reactions, Extractions, Chromatography and Archival. The user launches a program within Integrator and is presented with a series of menus specific to that program. This program then creates a command file. ACID then uses a single program. This program then creates a command file. ACID then uses a single program to read and interpret the command file for the Tecan. In the course of the execution of this program a log file and an error file are generated. Programs executing reactions also generate a tag file which describes the synthetic history of well locations. The tag file provides the links to the SD file containing structures.

### A fully automated stacking microplate luminometer for high throughput screening

**Steven Lenz, Ed Marples and Mel Reichman**
*Ligand Pharmaceuticals Inc., San Diego, CA, USA*

The Torcon Stacking Luminometer incorporates a Dynatech ML 1000 luminometer, reagent dispenser, computer, barcode reader and a plate stacking/shuttle device equipped with a mechanical gripper. The system is capable of reading stacks of up to 25 plates with a three fold increase in detection sensitivity. A plate is removed from the stack, placed on the plate shuttle and its barcode is read. The shuttle positions the plate under the reagent addition station, which is capable of dispensing between 10 and 300 µl of reagent. After reagent addition, the plate is shuttled to the luminometer loading position, where a robotic hand grips the plate and places it into the luminometer. The plate is read and the data analysed for out of range messages. If any are found, the gain is automatically adjusted and the plate read with the new gain. After the data is acquired the plate is placed into the output stack. The barcode on the plate is encoded with a unique identifier for storing the data and the default gain setting to be used by the luminometer. Data is written locally, then automatically uploaded to Oracle at the end of the run. The system failure rate is 0·1% of the plates processed and costs under $40K. ICN's plate stack magazines are used by the Torcon and can be interchanged with the stacking reader and platewasher devices used in the lab. This system will be modified for use with a CRS Robotics arm as part of a plate stacking robot system.

### Transferring and validating a biochemical assay into a high throughput screening assay

**Richard Harrison**
*Rhone Poulenc Rorer, Collegeville, PA, USA*

The drug discovery process is a multi-discipline, multi-faceted operation utilizing expertise from scientific backgrounds as diverse as genetics and chemistry. The process begins as biologists and geneticists discover new pathways and targets. Next, chemists synthesize small molecule inhibitors, agonists, or antagonists of these define targets. Finally, biologists and pharmacologists test these compounds in animal models for *in vivo* efficacy. Each step in the process is a transfer of information and technology from one scientific discipline to another. However, the process is a slow one, typically taking up to 10 years to identify and market a new drug entity. Historically, the slow step in the drug discovery process has been collecting and synthesizing compounds as potential drug candidates. With the advent of high speed parallel synthesis, a vast array of chemically diverse structures, previously unattainable, are created daily for biological characterization. Now, the slow step in the process becomes the characterization of compounds through biological assays.

Biological assays are generally crude systems designed to understand the workings of a protein or receptor, and for its initial characterization and purification. Such assays are inherently laborious, time consuming tests not generally suitable for screening a large number of compound variables such as cost reagent availability, reliability, limits of detection, and speed are not of paramount importance. Yet, these are the most important variables when designing a high throughput screening assay. It is therefore necessary to modify existing biological assays to fit in a high throughput environment. Still, no rules exist to guide this modification. This presentation focused on the steps used to transfer biological assays into a high throughput environment. The choice of assay, kinetic and thermodynamic characterization of the reagents, and assay validation were discussed. Examples of situations from the pharmaceutical industry were presented.

### Establishing a high throughput screening core group at Procter & Gamble Pharmaceuticals

**Jonathan Cook, Barbara Hynd, Barbara Kozikowski, Kathy Rasmusen, Deborah Paugh, Julie Whitten, Jennifer Sway**
*Procter & Gamble Pharmaceuticals, Cincinnati, OH, USA*

**Scott Atkin, Tim Bruemmer and Bryan Wildman**
*Sagian Incorporated, Indianapolis, IN, USA*

About two years ago the authors started assembling a screening group at Procter & Gamble Pharmaceuticals. The process was completed quickly and efficiently, and the group did not affect overall head count, although some personnel re-assignments occurred. In order to accomplish this, all expertise gaps in automation, database management, and screening facility operation have been filled through contracts and by consultation with recognized leaders. Automated compound dissolution and distribution, and a capability for both cell-based and biochemical assays are now in place. The group, located with the compound repository as one of its primary customers, has integrated software and tracking systems. Handling the high volume of data generated by HTS, associating results with the correct compound and integrating with company databases has been accomplished using CSAPTM from MLT Automation. The process of identifying and implementing the key components of the core facility formed the basis of this presentation.

### Use of a two armed Zymark robot for high throughput screening

**Mark Beggs**
*ZENECA Pharmaceuticals, Macclesfield, UK*

High throughput screening is a key component of the pharmaceutical lead identification process at ZENECA. Robotic systems have been employed throughout the industry to perform the initial compound dilution and distribution processes that form a common front end to many high throughput screens, and the assay assembly and signal detection steps that comprise an individual screen. Although the use of manipulative arm system in formulating and replicating test compounds is well established, robotic throughput rates have been significantly slower than those which can be maintained by human operatives and consequently the use of robots for performing assays has been limited. Over recent years the industry has experienced significant increases in the rate of compound screening and throughput rates are now approaching the upper limit that human operatives can be expected to achieve in a working day. To permit future increases in the rate of screening, a novel automation system was designed based on the use of two Zymark arms. The rationale behind automation of the screening process, the capabilities provided by the system and the resulting benefits were described.

### Automated high throughput screening to aid the drug hunter at GlaxoWellcome

**M. N. Banks, S. Fogarty, M. Valler, K. Mills, S. O'Brien, A. Binnie and J. G. Houston**
*GlaxoWellcome, Stevenage, UK*

Lead discovery within drug discovery at GlaxoWellcome's Medicines Research Centre plays a key role in the company's high throughput screening programme. This presentation described how automation has been used to facilitate this programme and the various approaches used. High throughput screening involves the testing of a range of different chemical entities in biochemical assays. Typically, these samples include single entity compounds, combinatorial compound libraries and partially or fully characterized natural products. The storage and preparation of these materials are performed by the Compound Diversity Unit and microtitre plates are provided for automated screening.

For the last two years a Robolab 9600 has been used, and recently a second screening system has been commissioned that will dramatically increase our high throughput screening capacity. This second system, R2, contains two robotic cells, one for performing isotopic assays and the second for non-isotopic work. This presentation described how this system was designed, built and commissioned to run on both 96 and 384 well microtitre plates.

### Developing smart robotic stations with embedded controllers

**Stephen Dokoupil**
*Helen Curtis, Inc., Rolling Meadows, IL, USA*

Often there is a need for the addition of extra sensors or control in a robotic station. At the same time the I/O available through the Zymate Power & Event Controller may be exhausted by existing system complexity. The use of simple embedded controllers or small single board computers can help expand the I/O capabilities of the robot system. A basic overview of embedded controllers was presented. This included the different types of controllers currently available, their cost and their use in a typical robotics or instrument application. Basic interfacing techniques and programming the controller were also covered.

### Creating reusable instrument objects with Visual Basic

**Martin Echols**
*SmithKline Beecham, Inc., King of Prussia, PA, USA*

**and Mark F. Russo**
*Bristol-Myers Squibb, Princeton, NJ, USA*

The relatively new object-oriented programming (OOP) paradigm has had a dramatic impact on software development. Yet, few examples of OOP applied to laboratory automation exist. Visual Basic 4.0 now supports many elements of OOP through the creation of objects. An object is a simple mechanism for encapsulating the properties and functions of a discrete software entity. The OOP approach can result in many benefits, including: an ability to easily reuse software modules, better debugging capabilities, and increased robustness.

Making use of OOP concepts for the development of an interface to a laboratory instrument is appealing. This 'instrument' object can be saved and easily re-used by other laboratory software applications. In this paper the authors discussed methods for creating objects for data acquisition and instrument control in Visual Basic 4.0. Strategies for managing and re-using user created instrument objects were presented, which enabled the creation of an instrument object library

An additional step in the development of a library of instrument objects is to reformat them as OLE (Object Linking and Embedding) objects. OLE is the foundation of the 'plug and play' concept that is currently popular in the computing industry. Methods for creating OLE objects from a library of instrument objects were also discussed.

### Visual basic applications in a laboratory environment

**Juan Cadavid and Marie Sabo**
*Clairol, Inc., Stamford, CT, USA*

In the last several years there has been a strong emphasis on doing things more efficiently. To accomplish this objective, the analytical group has been searching for productivity enhancement tools to apply in a laboratory environment. Small computer programs written in Visual Basic have been found to be one of those tools. These programs take advantage of the many capabilities of Visual Basic, especially its ability to create intuitive graphical user interfaces. One of the applications implemented was a program which predicts the final result of a titration based on the theoretical contributions of the different raw materials in a consumer product. This program is intuitive enough that the formulators can use it independently of their computer skills. The theoretical value obtained can also be used to verify the validity of the experimental results and to develop finished product specifications. Another important application implemented is capturing the data coming from a titration system and storing it in a database utilizing Visual Basic as the front end interface. This program allows the data to be reviewed at any time after the sample has been analysed.

The trials and tribulations of writing these computer programs were presented, as was the resulting impact of productivity.

### Reaction screening and optimization in chemical process research and development

**Barbara M. Ciaramella**
*Bristol-Myers Squibb Pharmaceutical Research Institute, New Brunswick, NJ, USA*

The shortening of drug development timelines has caused pharmaceutical companies to rethink their strategies for product development. One facet of Bristol-Myers Squibb's approach has been to incorporate robotics and automation into new discovery and development paradigms. Within the Process Research and Development department of Pharmaceutical Development, a robotic apparatus has been configured to run and monitor up to 16 reactions simultaneously, enabling faster data regeneration and reaction assessment, including route scouting and reaction optimization. This presentation discussed the hardware and custom software configuration and presented results to date.

### New approaches for material handling at Glaxo Wellcome

**Brenda Johnson Ray**
*GlaxoWellcome Inc., Research Triangle Park, NC, USA*

Chemical sample dispensing at GlaxoWellcome has undergone a major shift in recent years. Previously, solid sample weighing was one of the major functions of the Materials Management group, which has responsibility for the distribution of samples for discovery research. Now, high-throughput screening and combinatorial chemistry programs have been developed in order to synthesize and test more compounds, find better leads, and ultimately get a drug on to the market in less time. Consequently, the Materials Management Group has had to re-evaluate its role. First, the solid sample weighing bottleneck was eased with the routine provision of samples in liquid format. Next, repetitive manual steps were automated with stand-alone instrumentation or just simple changes in work practices. Finally, the Group evaluated options for linking the old solid handling practices to the new liquid handling procedures in order to transition smoothly from one to the other. The benefits and the process were described.

### Recent applications of accelerated structure analysis protocols

**Edward H. Kerns, Kevin J. Volks, Jeff L. Whitney, Robyn A. Rourick**
*Bristol-Myers Squibb Pharmaceutical Research Institute, Wallingford, CT, USA*

**Mark E. Hail and Mike S. Lee**
*Bristol-Myers Squibb Pharmaceuticals Research Institute, Princeton, NJ, USA*

Current trends in product research and development have focused on accelerated cycles (for example, preclinical development) and increased sample generation (for example, combinatorial chemistry). At the same time, resources for internal support of these initiatives using traditional paradigms have not increased at the same rate, leading to the necessity for a new paradigm. In this environment, mass spectrometry is the emerging analytical technology for meeting current and future needs, based on its sensitivity, selectivity, universal applicability, speed and cost-effectiveness. Integration of advanced mass spectrometry technology, development of new methodology and novel implementation strategies have provided increased levels of support and information generation consistent with current and future trends. These strategies, once developed, have been automated for optimum productivity. This presentation described recent development in structure characterization using molecular weight determination and in structure elucidation using LC/MS techniques. The role of standard methods, template structure analysis, predictive profiles, structure profile libraries and candidate analytical profiles were discussed. These were illustrated with recent applications from candidate proof of structure, combinatorial chemistry, natural product discovery, drug metabolism, impurities, and degradation products.

### An automated system for the purification of combinatorial libraries by semi-preparative HPLC

**Christopher E. Kibbey, Greg A. Robertson, Alasdair MacDonald and Sheila H. DeWitt**
*Parke-Davis Pharmaceutical Research, Ann Arbor, MI, USA*

Combinatorial chemistry has emerged as a powerful tool for the rapid generation of new pharmaceutical candidates. Improvements in laboratory robotics and information management systems have contributed to the field's rapid growth. While several systems are available for performing automated compound synthesis, there are comparatively fewer systems which automate the efficient purification of milligram quantities of compounds from combinatorial libraries. The high separation efficiencies afforded by commercial packed columns and level of automation inherent in modern liquid chromatographic equipment make HPLC well suited to the purification of combinatorial libraries. This presentation described an automated, semi-preparative HPLC system designed for the isolation of milligram quantities of single compounds from combinatorial arrays produced using solid-phase, or solution-phase techniques.

Purification of a sample library is performed in two chromatographic steps. First, a portion of the crude sample is chromatographed on an analytical-scale HPLC column and elution of the sample components is monitored by UV and mass spectrometric detection. The chromatogram from this scouting run is then imported into a custom program, where an intelligent peak tracking algorithm guides the isolation of selected product component(s) fom the crude sample by semi-preparative HPLC. Because positive identification of each component in the crude sample may be performed via an LC/MS scouting run, the system provides MS identification of the product component in the crude sample prior to chromatographic purification the need to collect and analyse multiple sample fractions during semi-preparative purification is eliminated. Further, the LC/MS data sets may be used to verify failed syntheses, and thereby speed the purification process by excluding these samples from further processing.

The purification system is compatible with both reversed-phase and normal-phase methods, and only requires that the same chromatographic support be packed in both the analytical and semi-preparative HPLC columns. Scaling a set of analytical chromatographic conditions to their semi-preparative equivalent is straight forward. The chromatographic methods employed may be tailored to meet the specifications imposed by the sample library. This becomes especially important when dealing with compound classes which are susceptible to hydrolysis, thermal degradation, or transesterification.

### Integrated drug discovery: thriving on organizational and technological improvements

**Peter Eckard, Juergen Delzer, Franz Emling, Siegmund Guhl, Reinhold Janocha, Claus Markert, Gerhard Paul, Juergen Seega and Wolfgang Weruet**
*Knoll AG, BASF Pharma, Ludwigshafen, Germany*

High throughput screening (HTS) has been established as a standard technology in the drug discovery process of BASF Pharma. With the HTS set-up presented at ISLAR 94, the authors have screened their chemical library in various enzyme or cell-based assays or ELISAs on different molecular targets. As a result, promising leads have been identified and some of these screening assays are now in an advanced stage of preclinical development. However, the routine use of HTS revealed several shortcomings which have been taken into account in a new version of the authors' HTS concept.

The demands on HTS have steadily been increasing over time. The number of compounds to be tested, as well as the number of targets to be screened, were boosted by combinatorial chemistry and Genomics respectively. In the decentralized HTS approach, where several laboratories in different units perform HTS and secondary screening, the HTS equipment could be improved. To match the needs resulting from these increasing demands and time constraints, technological improvements and organizational measures are mandatory.

The presentation described the authors' experiences of establishing HTS at Knoll AG and addressed the future perspectives covered by the new concept for integrated drug discovery (single compounds versus mixtures, preparation of samples, highly integrated robotic systems, HTS core unit versus decentralized approach and working time models).

### A component based approach to laboratory automation

**Mark N. Feiglin and Bruce J. Russell**
*Merck Research Laboratories, Rahway, NJ, USA*

While laboratory automation projects have grown in complexity, there has been a move towards using modular approaches in selecting instruments for automated systems. Rather than using a single complex instrument to perform a variety of tasks, a modular approach attempts to divide the tasks among a number of specialized instruments. Each instrument is often designed for a specific role and is able to work independently of the other instruments on a system. In this manner, the failure of one instrument does not necessarily mean the collapse of the entire system. A modular system also follows for easy modifications to the system as needs change.

During the authors' work in laboratory automation, they have further developed this concept. In addition to using modular instrumentation, a design has been developed that also stresses a modular approach to software design. In designing a modular software approach to laboratory automation, it is important to select an operating system that is flexible, robust, secure, and itself modular. One such operating system is Microsoft Windows NT.

The true pre-emptive multi-tasking provided by Windows NT prevents the collapse of one piece of software from affecting others controlling an automated system. The built-in security features of Windows NT allow the administrators of a robotics system to prevent the users from accidentally deleting files and data important to the operation of the system. Windows NT also allows a degree of flexibility and modularity unavailable in most operating systems. Computer hardware devices (such as multi-port serial cards and IEEE cards) are handled by the operating system rather than separate device drivers. This allows for software design that is truly hardware independent. Windows NT offers some additional benefits, including a limit of 256 serial ports per computer, remote system maintenance, and increased system resources.

Designing a robotics system on an object-based operating system allows the use of object based programming languages to develop software tools for automation. When defining a component in a robotics system, it can include both the instrumentation and software required for its operation. Object based programming languages such as Microsoft Visual Basic 4.0 allow for a modular approach to instrument control. The instrument control software can also be used without the automated system. This allows the users to become used to the look and feel of the instruments integrated with their software drivers in a stand alone mode. The future of modular laboratory automation includes a modular software approach, as well as modular instrumentation. The authors have begun development of such a system and presented some of their experiences.

### High throughput screening through the use of robotics and a high performance data handling system

**Shin-Ichi Nihira, Hiromichi Kotaki and Atsuko Nakano**
*Nippon Roche Research Centre, Kamakura, Kanagawa, Japan*

High throughput screening (HTS) using various automated devices including robots has expanded the capabilities of random screening of natural products and chemical libraries for drug discovery during the past several years. It is a promising approach in the rapid and efficient identification of compounds. The utilization of robotics coupled with a high throughput data handling system allows hundreds of thousands of screening samples (natural products as well as synthetic chemical compounds) to be processed in a realistic screening period. For further improvement in performance of the entire process of HTS, the following issues must be addressed: logistics of assay samples; informatics; screening automation.

The authors' group is responsible for HTS and for implementation of HTS related technologies. During the past few years, the screening process has been developed to accommodate extremely large number of samples derived from chemical compound libraries and natural products.

This presentation focused on the authors' practical experience and presented some issues related to interfacing of both systems.

### Automation of assays for nuclear receptors

**Steven G. Blanchard, Cole O. Harris, James S. Nichols and Derek J. Parks**
*GlaxoWellcome Inc., Research Triangle Park, NC, USA*

The development and automation of functional and ligand binding assays for members of the steroid/thyroid superfamily of nuclear hormone receptors was described. The use of stand-alone instrumentation and assay streamlining allowed for rapid automation without any resulting delay. The assay formats utilized are generic and have been verified for multiple nuclear receptors. As a result, implementation of additional assays for members of the nuclear receptor superfamily can be accomplished with minimal assay development.

### Implementation of the TPW II Powder Pouring Workstation for on-line analysis of process validation samples

**Thomas D. Groeschner**
*Schein Pharmaceutical, Inc., Carmel, NY, USA*

The current analysis of process validation samples by current manual analytical procedures has demonstrated the need for automation due to the large sample load and the labour intensive nature of the manual preparation procedure. At Schein Pharmaceutical, the idea of automating the procedures to analyse process validation samples (powdered samples) was taken to Zymark Corporation. In conjunction with Schein Pharmaceutical, a TPW II workstation was developed to handle all the parameters required to process powdered process validation samples.

Schein demonstrated automation for a number of reasons. First, the FDA requires that all of the contents of a process validation sample be analysed. To accomplish this manually, the bottle must first be weighed, the contents of a bottle must then be emptied and the bottle rinsed several times. Then, after drying, the bottle must be reweighed to obtain the sample weight. This process takes one analyst approximately 30 min per powdered blend sample. Second, the large number of powdered samples associated with a process validation (20–50 blend samples) is extremely labour intensive and requires 10 man hours to prepare only 20 powdered samples. Third, in the generic industry there is a high demand for maximum throughput with minimum manpower. Therefore the need was to increase the efficiency of the testing of process validation samples and it was determined that automation was the avenue to take to accomplish this goal.

This presentation described the requirements of a system to handle powdered process validation samples. The benefits of an automated procedure as opposed to a manual procedure were explained.

### Experiences with fibre optic technology in automatic dissolution testing

**H. D. Friedel and D. Wortig**
*Bayer AG, Leverkusen, Germany*

In recent years, dissolution testing has become increasingly important in the development of new solid dosage forms. Dissolution results also indicate batch homogeneity and conformity. Samples are generally removed from the dissolution vessel through a filter at specified intervals. The active ingredient content is determined by HPLC or UV spectroscopy, both with manual sampling and in automatic systems.

Precipitation or adsorption of the drug on the filters or tubes may occur when the sample is taken. The amount of released active ingredient is best determined ***in situ*** directly in the vessel. The reading is taken very quickly, enabling release kinetics from very quickly releasing tablets to be recorded. A direct measurement is carried out using a dipping probe connected via a fibre optic cable to a UV/VIS spectrometer. The detector is integrated into an automatic system. A robot, manufactured by Zymark, moves on a linear axis and dips the measuring probe in the solution at the times stipulated. The system carries out dissolution tests fully automatically. All steps are automated, from preparing the media, filling the dissolution vessels and adding the tablet, to calibration, measuring, and cleaning the vessels. Robot systems with fibre optic detection, which allow several tablet batches to be analysed without supervision, have been working in the authors' laboratory since 1992.

### A QA robotic system to perform chemical analyses

**Marie Sabo and Juan Cadavid**
*Clairol, Inc., Stamford, CT, USA*

A robotic system has been designed for and implemented in the chemical quality assurance laboratory, which supports consumer product production operations. The initial decision was made to automate chemical testing of the largest volume manufactured products, both as in-process and final package samples, which are in five aluminium tubes. A proposal was presented for a Zymark custom-designed Zymate robotic system capable of addressing the highest throughput analyses performed: two titrations (acid-base type) and a viscosity measurement. These titrations have historically been performed manually in QA, although Analytical had used Brinkmann autotitrators for some time. Since the chemistry was well known and the autotritrator methods had already proven to be accurate and precise, these appeared to be ideal analyses for robotic automation. The viscosity determinations would not be as straightforward; since the samples had only been prepared manually no automation had yet been developed. It was felt that the sample preparation could be automated with some effort, and in fact, would probably be more reproducible than the manual procedure. The actual viscosity measurement is made using a Brookfield viscometer. The system would also need to contain and dispense at least 10 different reagents and be able to move, manipulate, and clean-up materials with a range of viscosities from water-thin to thick gels.

Although it would initially be used by QA analysts, the system was designed with a non-scientist operator in mind. The long-term goal is for a production worker to be able to bring a sample to the robot, be computer prompted through sample entry into the sytem, and some time later receive an 'approved' or 'not approved' result. (The latter situation would then require QA-human intervention.) It was, therefore, necessary to develop a database of all products, product specifications, test methods, method parameters, variables, and calculations for incorporation into the system. The user should only need to know and enter two pieces of information into the system: a product number and whether the sample is in-process or in a final package. The computer should tell the operation exactly where to place the sample in the system racks. The database would need to contain information and specs for over 500 products to be analysed using about 30 method variations.

The robotic system which meets these requirements was presented and discussed. Recent enhancements made to the original operation design, which have expanded the use of the system, were also described.

### It's 1.00 a.m.—do you know how your robot is performing?

**J. R. Ormand**
*The Dow Chemical Company, Midland, MI, USA*

For several years, the author's laboratory has been focusing on improving the efficiency of its work processes. A key measurement is cycle-time for sample sets. Large cycle-time reductions have been achieved through standardization of sample preparation, analysis, data reduction, report writing, and data review. Further reduction of sample set cycle-time has been investigated by examining the effectiveness of the automated systems.

This presentation described the performance of a Zymate XP robot which has been used for the five years to perform a complex liquid/solid extraction process. The system's performance has been studied for approximately two years, focusing on the number of sample preparations that are completed without operator intervention. The details of these measurements, comparisons to other applications and systems, as well as the implementation of process improvements were discussed.

### Application of automated polymer analysis

**Paul L. Morabito, John J. Zieman, Ramasamy Tamilarasan, Ward L. Rigot**
*The Dow Chemical Company, Midland, MI, USA*

**Paul D. Hazelwood, Mark Cranch and Roger Gagnon**
*The Dow Chemical Company, Samia, Ontario, Canada*

Over the past three years, laboratory robotic automation efforts within Dow Chemical have been directed towards developing systems to prepare a variety of different polymer samples for subsequent analysis. These systems required development of custom hardware modules to provide automation for both high and low temperature dissolution techniques, for solution filtration and concentration techniques, analytical instruments interfaces and multi-method capabilities. Because of the complexity associated with the polymer preparation and lack of suitable on-line analysers, laboratory robotics were chosen as the automation platforms. Recently, two robotics automation systems were developed and installed in the high Density Polyethylene and Polystyrene Samia production facilities. This presentation described the technical merits of the hardware automation, as well as the complex flexible operating protocols required to rapidly support production.

### The care and feeding of robot Jock

**Norman E. Fraley, Jr.**
*Kelly Scientific Resources, Des Plaines, IL, USA*

This presentation took a light-hearted look at the issues involved with robots and personnel. The introduction of the robot tends to generate the amazing transmogrification of normal chemists into the robot Jock. This new species of scientist has its own unique set of needs and feeds. This creature was examined both in the wild and in its natural habitat. Some of the fears and favours of this entity were explained and ways to ensure that this rare, and extremely valuable, creature is well cared for were outlined.

### Open access mass spectrometry in combinatorial chemistry

**Lester C. Taylor, Jeremy Batt, Robert L. Johnson and Melanie Traynor**
*GlaxoWellcome, Research Triangle Park, NC, USA*

The introduction of open access mass spectrometry has significantly changed the way in which the synthetic chemist undertakes routine sample analysis. The availability of walk-up instruments allows the chemist to carry out sample analysis usually within a few minutes. As a result, mass spectrometry is used routinely for reaction monitoring, analysis of analytical and preparative HPLC fractions, synthetic intermediates and final products. The development of atmospheric pressure ionization (API) has facilitated routine and reliable operation of such general access instruments. This includes the use of electrospray (ES) and atmospheric pressure chemical ionization (APCI) sources which provide molecular weight information (often with some degree of fragmentation) for the vast majority of polar, thermally labile molecules. However, there are compound classes (for example, less polar, volatile compounds) which do not give molecular weight information by API and for which chemical ionization is a more appropriate ionization mode. As a result the authors have also introduced an Open Access GC-MS instrument for the analysis of such compounds.

Combinatorial synthesis is being adopted as a routine approach for the generation of large numbers of compounds in the drug discovery process. This has placed an increased burden on analytical methods to support characterization and synthesis validation. A mass spectrometer has been acquired which utilizes a Gilson 215 autosampler directly sample 96-well microtiter plates at the rate of approximately one sample per minute. This system utilizes computer software to facilitate data analysis and rapidly validate the chemistries for each plate. Target compound molecular weights can be searched and compared automatically to the data for each well analysis, providing the chemistry with a quick evaluation of library synthesis.

### Adding value to enterprise-wide research and development programs through automated NMR spectroscopy

**William C. Hutton**
*Corporate Research, Monsanto Company, St. Louis, MO, USA*

NMR spectroscopy generates information vital to businesses who use chemistry and biology to invent and develop new products. NMR spectroscopy is resource intensive as well. Laboratory automation brings value by maximizing the quantity and quality of NMR data throughput and R&D enterprise. This paper presented a unique robotics system used to collect NMR data. The approach provides the efficiency of automated measurement without sacrificing flexibility. The impact on the NMR laboratory as well as the technical community were addressed. The technical and cultural obstacles encountered during this project were discussed. Information technology is no less important than productive measurement of spectra. An NMR information system based on a UNIX/TCP-IP/X-Windows computer network was presented.

### Automation of structure analysis in pharmaceutical R&D

**Gary A. McClusky, Brian Tobias, Robert Short, Dana E. Dejohn, Leslie McMacken, Carmen Faraianu and Bryan Judge**
*Parke-David Pharmaceutical Research, Ann Arbor, MI, USA*

The analytical chemistry laboratory in pharmaceutical research continues to be challenged to perform structural characterization rapidly and accurately. This is fuelled by improvements in organic synthesis, such as automated synthesis and combinatorial chemistry, and the availability of better structural tools and methods. Advances in instrumentation, robotics and computer technology have enabled automation of the associated tasks, such as sample preparation, sample introduction, data acquisition and procession, and data storage and retrieval. The integration of these components with laboratory information systems results in improved overall efficiency of laboratory operation. This, in turn, allows the laboratory analyst to direct significant time to challenging structural characterization projects and away from standard analysis.

Laboratory robotics have been used to automate sample preparation for NMR and MS analysis. Barcoded sample vials are delivered to the robot and are prepared using information obtained from the laboratory information management system (LIMS). Modules associated with this system include barcode reader, solvent dispensing station, vortex mixer, pipetter, and turbidity check and filtration stations. The dissolved sample solution is dispensed into an NMR tube or MS vial, which is then capped, and placed into an autosampler rack on the respective spectrometer.

Some analysis is performed in a batch mode on automated NMR and mass spectrometers using instrument worklists download from the LIMS. Data acquisition and processing occur without analyst intervention. Data file transfer and loading completed results into the LIMS are triggered by the analyst.

Data files are archived on a file server equipped with an optical jukebox and indexed in a relational database. Data files are retrieved through querying the database and transferring files back to a spectrometer. Information system features include sample login front-end for customer use, e-mail notification of results, intranet access to sample and instrument status, and results through Netscape. The careful integration of analytical instrumentation, robotics and information systems results in an efficiently operating laboratory that can meet the ever increasing demands of a research environment.

### High throughput cell based assay robotics system with integrated diagnostics and data management

**Steven Lenz, Ed Marples and Mel Reichman**
*Ligand Pharmaceuticals Inc., San Diego, CA, USA*

Cell based assays are performed in a high throughput mode for drug screening at Ligand. A robotics system for automatically performing these assays in the evening, and functional as a series of independent workstations for use during the day, was built and implemented. The system integrates a 96-well pipettor, plate washer, reagent addition station, luminometer and a tissue culture incubator with a CRS Robotics A265 arm and flexible data management software by MLR Automation. The 96-well pipettor is a Robbin's Hydra that was modified with a wash station and plate shuttling device. It is used to pipette 1 μl of compound directly into the assay plates at better than 7% CV. The incubator was modified with miniature pneumatically actuated doors and rotating 120 plate carousel. These modifications and the slowly rotating carousel, minimize environmental disturbances and edge effects caused by the robot accessing the incubator. Reagents, compound and assay plates are barcoded with unique identifiers. This information is read by the robot and written into the data file headers. MLR Automation's CSAP software has been modified to interface with Oracle. It automatically processes and uploads the data based on the file headers. CSAP then generates a list of retests to be submitted to the compound handling system. A vision system and modem have been integrated into the system's computer. This allows the robot or an operator to transmit still photos of the robot when the system halts due to an error. The operator may then remotely diagnose the problem and attempt corrective action.

### High-throughput screening by remote control

**Junko Aimi and Michael R. Kozlowski**
*Geron Corporation, Menlo Park, CA, USA*

Laboratory automation is an essential component of the high-throughput screening laboratory. The robotics systems are used to perform repetitive functions and free humans for more creative activities. Major limitations of automated systems are: errors occur during the course of a large run; software programs available for performing high-throughput screens are not flexible enough to accommodate special needs within an assay; and changes need to be implemented in a timely manner.

This presentation discussed approaches designed for monitoring automated screens and for performing real-time modifications of protocols and error corrections. This included a simple baby-sitting program that contacts individuals when an interruption in an automated run is detected; a video monitoring system that allows an individual to survey multiple systems and/or supervise robotics operations from a remote location; and an interactive system (under development) which allows monitoring of robot modules and error corrections in real-time.

### Automated systems for dilute solution viscosity measurement of polymers using a Zymate Robot and a Viscotek Differential Viscometer

**Sador S. Black, Tab Crawford and Harold Kinder**
*Eastman Chemical Company, Kingsport, TN, USA*

Solution viscosity analysis is a fast, simple, and reliable method for determining the molecular weight of a polymer. The polymer molecular weight is an important property since it reflects the ability of the polymer to be processed and the polymer's fitness for use in various applications. In the Polymers Division of Eastman Chemical Company, the Analytical Services Laboratory is responsible for determining the Inherent Viscosity (IV) of all production process samples. IV determination can be broken down into several steps: sample preparation; sample analysis; sample cleanup; and data reporting. Each of these steps was previously performed by laboratory analysts in the Analytical Services lab. The motivation to automate IV testing came from several sources. The first incentive was an increasing sample load. It was believed that automation, coupled with the use of different viscometers, could increase laboratory sample capacity. Safety was another incentive. The solvent which is used to dissolve the polymer sample is very hazardous. Automation was expected to greatly reduce the analysts' exposure to the solvent. There was also an economic incentive. Two analysts were required to perform the manual IV test. It was felt that automation of the system would reduce this number to one and free the second analyst to perform other jobs.

The process of transferring the manual operation to an automated one required hardware and software integration between the Zymate robot, the PC used to control the differential viscometer, the LIMS computer in the Analytical Services Lab and the Polymers Manufacturing Information System (a VAX computer which maintains all production data). The integration process required many trials, and custom equipment was needed for several robot functions. The current system consists of two Zymate XP robots performing solution viscosity analysis on a 24 hour basis, 365 days per year.

### To buy or not to buy? Peripherals from robot suppliers

**Michael R. Kozlowski**
*Geron Corporation, Menlo Park, CA, USA*

High-throughput screening groups are frequently faced with difficult decisions concerning the most effective ways of automating drug discovery processes. These decisions generally do not involve which robotic arm to use, since the number of choices in this area is still relatively small, but the type of instruments that the robotic arm will serve (peripherals). There are two broad types of robot peripherals. The first are stand alone instruments—these are often automated but are not integrated with a robotic arm. The integration is left as an exercise for the purchases. The second class is integrated instruments. These are designed to be used with a robotic arm (often exclusively) and are available from suppliers of robotic arms as modules or as part of a turn-key robotic platform. Each of these types of peripherals has its strengths and weaknesses, some of which are related to the type of task being performed, as well as the laboratory environment. Because of the time and expense involved in setting up a robotic screening laboratory, an incorrect choice of robot peripherals can cause, for the laboratory manager, considerable loss of efficiency, face, and often another anatomical feature. This paper discussed options in choosing robot peripherals, and how to select the correct instruments.

### Robotic control of whole blood processing in a clinical laboratory for functional brain imaging

**David L. Alexoff, Payton King and S. John Gatley**
*Brookhaven National Laboratory, Upton, NY, USA*

Clinical and basic research protocols using functional imaging techniques like Positron Emission Tomography (PET) or Single Photon Emission Tomography (SPET) often require a significant personnel commitment to the sampling and processing of whole blood samples from human and non-human primate subjects. Blood gas, plasma glucose, and radioactivity concentrations must be determined for each imaging study. Faced with decreasing research operating budgets and the anticipated addition of a second tomograph, the authors' laboratory has begun investigating the utility of using laboratory robots to carry out some of these routine clinical laboratory tasks necessary for clinical and basic research protocols using PET.

This presentation described the routine clinical laboratory tasks needed in PET research and how these tasks have been automated using a commercially available laboratory robot system. The system is based on standard components of Zymark Corporation's (Hopkinton, MA, USA) PyTechnology systems. No custom robot hardware was used, and all software was developed at Brookhaven

National Laboratory. An algorithmic approach to solving the problem of pipetting plasma from small-volume whole blood samples (< 0·04 ml) was described. The system automatically centrifuges small-volume whole blood samples, pipettes and weighs plasma, and assays 511 KeV photon radioactivity in the plasma. Steady state robotic throughput is 143 samples/hour for auto-sampled whole blood. Results and performance of the robotic system were compared with those obtained manually by an experienced laboratory technician.

This research was carried out at Brookhaven National laboratory under contract DE-AC02-76CH00016 with the US Department of Energy and supported by its Office of Health and Environmental Research and also supported by the National Institutes of Health Grant NS-15380.

### It's only a matter of time

**F. H. Zenie**
*Zymark Corporation, Hopkinton, MA, USA*

Columbus set out for China and discovered America. Was the journey a success or failure? It has to be a failure unless you redefine the goals. We are surrounded by opportunities, the challenge is recognizing them and then redefining our goals to capitalize on the new opportunity.

Changing markets and economics demand business innovation. We are learning that new technologies enable change, but management must lead business innovation. Today's managers must shift from directing work to translating business strategies into action plans and energizing people to implement the plans. Simplistic accounting measurements are being replaced by business impact analysis, and judgment.

Over the past 15 years, laboratory automation has grown from a novel new technology to a powerful management tool to help leading organizations become more innovative, productive and competitive. The greatest value comes from applying automation in the context of business strategies. Those who excel will gain competitive advantage, grow and prosper.

### The use of robotics in a cosmetic microbiology laboratory

**Steven F. Schnittger**
*Estee Lauder Companies, Melville, NY, USA*

The preservative challenge test is a method used to determine the efficacy of a preservation system in a cosmetic formulation. The method of testing is a labour intensive, repetitive task. Product testing entails a large volume of samples which are analysed repeatedly under the same conditions and protocol. For this reason, an automated robotic system was developed to perform this testing and to free the cosmetic microbiologist to perform more meaningful and creative tasks.

Two hundred and fourteen different formulations totalling 1039 samples were evaluated, comparing the automated robotic system to the manual plate count method of testing. The samples comprised make-ups, shampoos, conditioners, oil in water emulsions, mascaras and powders. The selection of micro-organisms tested are similar to those recommended by the CTFA Guideline for the Preservation Testing of Eye Area Products.

The results showed that there was a greater than 98% correlation in results when comparing the automated plating system to the manual method of testing. It has also been determined that the robot can save between two to three man hours per day.

The unattended sample preparation of the robot, allows for testing to continue beyond the normal work day of the job. By programming the robot to run 24 hours per day, the microbiologist or technician is able to perform other functions in the lab. It also allows for an increase in the workload of the lab, improving the throughput of the lab, without additional personel.

